# Retina regeneration: lessons from vertebrates

**DOI:** 10.1093/oons/kvac012

**Published:** 2022-08-02

**Authors:** Poonam Sharma, Rajesh Ramachandran

**Affiliations:** Department of Biological Sciences, Indian Institute of Science Education and Research, Mohali, Knowledge City, SAS Nagar, Sector 81, Manauli PO, 140306 Mohali, Punjab, India; Department of Biological Sciences, Indian Institute of Science Education and Research, Mohali, Knowledge City, SAS Nagar, Sector 81, Manauli PO, 140306 Mohali, Punjab, India

## Abstract

Unlike mammals, vertebrates such as fishes and frogs exhibit remarkable tissue regeneration including the central nervous system. Retina being part of the central nervous system has attracted the interest of several research groups to explore its regenerative ability in different vertebrate models including mice. Fishes and frogs completely restore the size, shape and tissue structure of an injured retina. Several studies have unraveled molecular mechanisms underlying retina regeneration. In teleosts, soon after injury, the Müller glial cells of the retina reprogram to form a proliferating population of Müller glia-derived progenitor cells capable of differentiating into various neural cell types and Müller glia. In amphibians, the transdifferentiation of retinal pigment epithelium and differentiation of ciliary marginal zone cells contribute to retina regeneration. In chicks and mice, supplementation with external growth factors or genetic modifications cause a partial regenerative response in the damaged retina. The initiation of retina regeneration is achieved through sequential orchestration of gene expression through controlled modulations in the genetic and epigenetic landscape of the progenitor cells. Several developmental biology pathways are turned on during the Müller glia reprogramming, retinal pigment epithelium transdifferentiation and ciliary marginal zone differentiation. Further, several tumorigenic pathways and gene expression events also contribute to the complete regeneration cascade of events. In this review, we address the various retinal injury paradigms and subsequent gene expression events governed in different vertebrate species. Further, we compared how vertebrates such as teleost fishes and amphibians can achieve excellent regenerative responses in the retina compared with their mammalian counterparts.

## INTRODUCTION

The regenerative capacity varies remarkably among animals. Mammals often regenerate a limited set of tissues and organs such as the skin, liver, epithelia of kidney, gut and lungs [1–5] and often fail to regenerate complex organs such as the brain, retina or heart. In contrast, the non-mammalian vertebrates such as teleost fishes and amphibians exhibit a robust regenerative capacity even in complex organs after remarkable tissue damage [6, 7]. The teleost model, zebrafish, is extensively used to study organ regeneration, especially the retina [8, 9]. The retina is the neurosensory part of the eye, which originates as an outgrowth of the diencephalon and is thus a part of the central nervous system (CNS). CNS has the least regenerative potential in mammals and hence their retina cannot proceed for successful regeneration. Major debilitating eye diseases like age-related macular degeneration, glaucoma and diabetic retinopathy cause visual impairment due to the loss of retinal neurons. Retina regeneration studies include fish, newts, frogs, embryonic chicks and rodents. It is intriguing to explore the potential of the retina to regenerate after an injury in humans, which could be helped by lessons from cold-blooded vertebrates such as fishes and frogs.

Soon after a retinal injury the Müller glia of the retina reprogram giving rise to a proliferating population of retinal progenitors (Müller glia-derived progenitor cells, MGPCs) that eventually replace the damaged retina with functional retinal neurons and Müller glia. Here, various retinal injury paradigms and molecular mechanisms leading to regeneration in lower vertebrates are compared to chicks and mammals. The cascade of events during retina regeneration is also explained in comparison to higher vertebrates while drawing parallels to developmental biology and cancer. The regenerative capability of primitive vertebrates is significant and complete, probably because of their ability to prevent infection in the aquatic environment and resort to slow regeneration compared to faster wound healing. The terrestrial animals rather indulge in a faster wound healing soon after injury, unlike their aquatic counterparts. The reasons behind this contrasting approach hold the key to robust regenerative capability in non-mammalian vertebrates. Based on the available literature, here, we try to comprehend and contrast the mammalian and non-mammalian approaches to retina regeneration and highlight the benefits imparted during mammalian retina regeneration while adopting gene expression paradigms from non-mammalian vertebrates.

## INJURY PARADIGMS

**Figure 1:**
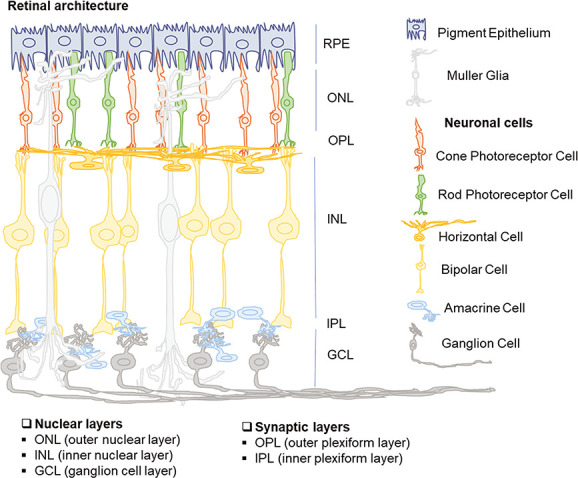
A diagrammatic representation of retinal architecture. The innermost layer facing the vitreous is the ganglion cell layer (GCL), comprising cell bodies of ganglion cells whose axonal extensions give rise to the optic nerve. The GCL is connected to the inner nuclear (INL) via the inner plexiform layer (IPL), where cytoplasmic extensions of ganglion cells connect with bipolar cells via amacrine cells as interneurons (amacrine cells shuffle between INL and GCL). The INL has three neuronal cell types (amacrine cells, bipolar cells and horizontal cells) and one non-neuronal cell, Müller glia. The INL is connected to the outer nuclear layer (ONL) via another synaptic layer, the outer plexiform layer (OPL). The cytoplasmic extensions of bipolar cells are connected to photoreceptor cells with horizontal cells acting as interneurons. The rod and cone photoreceptor cells constitute the ONL, the outer fragment of whose is covered with RPE.

**Figure 2:**
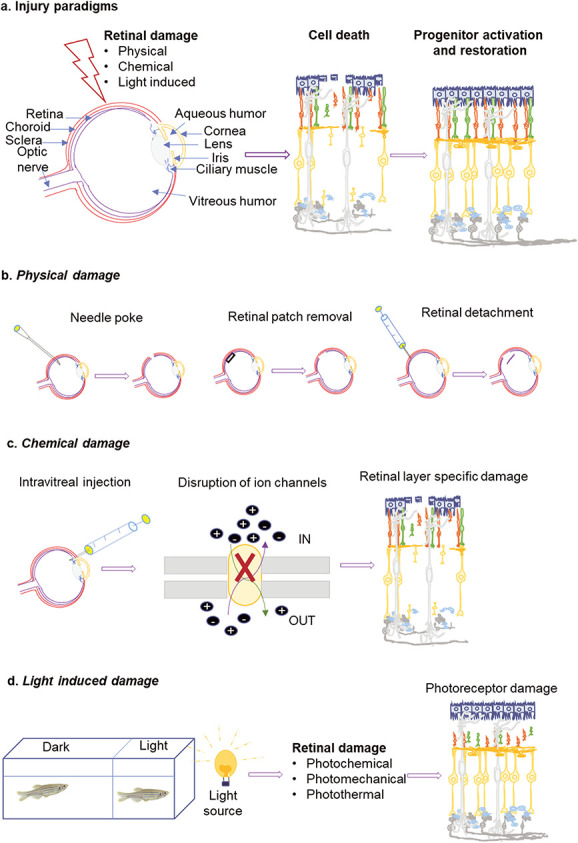
A pictorial representation of different retinal damage methods. (a) The different parts of the eye are shown along with three primary mechanisms inducing damage in various retinal cell types, which lead to restoration in regeneration competent animals like zebrafish or embryonic chicks and amphibians. (b) The three different modes of physical damage involve injury mediated with a 30G needle, removal of a small retinal patch with a micro knife and subretinal injection to induce retinal detachment. (c) Damage to different retinal layers caused by intravitreal injection of varying concentrations of chemical agents. These chemical agents cause cell death by disrupting the ion channels. (d) The damage to photoreceptor cells was induced after keeping the fish in the dark chamber for a long time followed by short-term exposure to intensely bright light. Light exposure causes cellular damage through chemical, mechanical or thermal mechanisms.

Retinal damage happens by various mechanisms that target either the whole retina or specific retinal cell layers. The different methods of inducing retinal injury include mechanical, chemical, light-induced or genetic ablation in transgenic animals (Figs 1 and 2; Table 1).

**Table 1:** The injury paradigms used in different animal models along with the retinal cell types or layers affected

Animal	Injury paradigm	Cells damaged
Fish	**Mechanical**	
	*Cryoinjury* [206]	All the retinal cells
	*Needle poke* [26]	All the retinal cells
	*Surgical excision* [29, 30]	All the retinal cells
	**Chemical**	
	*ATP* [207]	Photoreceptors, ganglion cells
	*Ouabain* [32–35]	GCL, INL or ONL
	*NMDA* [39, 41, 85]	GCL
	*MNU* [40, 208]	Photoreceptors
	6-*OHDA* [209–211]	Dopaminergic neurons
	*Tunicamycin* [212]	Photoreceptors
	*CoCl2* [52–54]	Photoreceptors, ganglion cells
	**Light-induced**	
	[56, 57, 85, 213, 214]	Photoreceptors
	**Genetic ablation**	
	*Nitroreductase/metronidazole* [215–218]	Photoreceptors, bipolar cells
	*Retinitis pigmentosa* [219, 220]	Photoreceptors
	*Diabetic retinopathy* [221–223]	RGCs
	*Age-related macular degeneration* [224–226]	RPE and photoreceptors
	*Glaucoma* [227, 228]	RGCs
Amphibians	**Mechanical**	
	*Surgical removal* [69, 70]	All the retinal cells
	**Chemical**	
	*Tunicamycin* [46]	Photoreceptors
	**Genetic ablation**	
	*Nitroreductase/metronidazole* [229, 230]	Photoreceptors
	*Retinitis pigmentosa* [231–233]	Photoreceptors
Birds	**Mechanical**	
	*Surgical removal* [73–75, 160] **Chemical**	All the retinal cells
	*NMDA* [80, 89, 234]	GCL
Mammals	**Mechanical**	
	*Retinal detachment* [27, 28]	Photoreceptors
	**Chemical**	
	*NMDA* [37, 38, 40, 42, 90]	GCL
	*MNU* [40, 235]	Photoreceptors
	*Tunicamycin* [48]	Photoreceptors
	*Ouabain* [236]	INL
	**Light-induced**	
	[237, 238]	Photoreceptors
	**Genetic ablation**	
	*Retinitis pigmentosa* [239, 240]	Photoreceptors
	*Diabetic retinopathy* [241, 242]	RGCs
	*Age-related macular degeneration* [243, 244]	RPE and photoreceptors
	*Glaucoma* [245–248]	RGCs

Mechanical injury is one of the feasible methods to study whole retina regeneration that ensures uniform damage to all retinal layers [10]. It involves surgical procedures, including a poke, small incisions or even the removal of a small retinal patch. In poke or stab wound injury with a 30G needle, the eyeball is tilted with the help of forceps and stabbed with a needle so that it penetrates all the retinal layers to the vitreous [11]. Another injury method involves retinal detachment with subretinal saline or hyaluronic acid injections through small scleral incisions [12, 13]. Retina regeneration studies also involve the removal of a small retinal flap, including all retinal layers involving transscleral injuries with the help of a micro-knife and the removal of a small rectangular retinal patch [14, 15]. In general, mechanical damage is a frequent mode of injury to animal tissue. Repairing a mechanical injury through regeneration or wound healing could have been reflected in the natural selection and evolutionary advantage to the animal’s survival.

The chemical injury involves injecting chemical moieties such as ouabain, N-methyl D-aspartate (NMDA), 6-hydroxy dopamine (6-OHDA), tunicamycin, N-methyl-N-nitrosourea (MNU) or hypoxia inducing factors. These chemicals are injected in varying doses depending on the retinal layer to be targeted and hence can be used to mimic various eye diseases. Ouabain is a cardiac glycoside that inhibits the Na+/K+ pump by binding to the K+ binding site of ATPase, thus affecting the resting potential of nerves and disrupting the retinal metabolism [16]. Varying concentrations of ouabain have a differential effect on retinal layers [17, 18], whole retina [19], ganglion cell layer, inner nuclear layer [20] and photoreceptor cells [17]. NMDA causes neuronal cell death by overexciting synapses due to increased NMDA receptor-mediated cation influx [21]. NMDA has been used to cause retinal injury by inducing ganglion cell death, activating glial cells to cope with damage [22–27]. The most accessible dopaminergic neurons of the vertebrate retina are dopaminergic amacrine cells. Retinal dopamine has multiple roles in vision, like adapting light/dark retinal circuits and influencing trophic processes [28]. The 6-OHDA (a hydroxylated analog of natural dopamine) is one of the most common neurotoxins that induce rapid death of dopaminergic neurons. In the solution, 6-OHDA converts to quinone, which stimulates the production of free radicals. For using relatively high doses of 6-OHDA, sodium ascorbate in the solution prevents the free radical formation and hence non-specific damage [25, 44]. Tunicamycin is an antibiotic inhibiting N-glycosylation of asparagine-linked oligosaccharides [45]. Rod photoreceptors contain the visual pigment rhodopsin, a membrane protein with two asparagine-linked glycoproteins. Tunicamycin treatment inhibits glycosylation of opsin, leading to disruption of the rod outer segment membrane assembly [31] and shortening of rod outer segments and hence photoreceptor-specific damage [47, 48]. MNU is an alkylating agent causing DNA damage. It causes photoreceptor damage by apoptosis-induced cell death [49]. CoCl_2_ affects hemoglobin by preventing iron inclusion in heme and affecting oxygen carriage, leading to the production of hypoxia-inducible factors [50]. CoCl_2_ also inhibits proteasomal degradation of hypoxia-inducing factors, thereby promoting hypoxia-mediated injury [36]. Intravitreal injection of CoCl_2_ is used to mediate hypoxic injury and has been used to damage different retinal layers as photoreceptors [52, 53] or ganglion cell layers [54]. Chemical modes of retinal injury enable us to understand and emphasize the regenerative mechanisms adopted by an organism in response to various cellular insults initiated by chemical toxins.

Light is necessary for vision, but constant long-term exposures or high-intensity light can damage the photoreceptor layer [55]. The electromagnetic spectrum in the range of 400–1400 nm, which is allowed to pass through the retina, is considered the retinal hazard zone [55]. Light-induced damage follows the disruption of the standard light and dark cycle to a long dark cycle followed by exposure to high-intensity visible light [56] or ultraviolet light [57]. The light-induced damage is with tungsten halogen lamps, metal halide lamps and fiber optics [25]. Photothermal, photochemical and photomechanical mechanisms can mediate light-induced damage. Photothermal damage happens by the transfer of radiant energy in the form of photons to the retinal tissue, which increases intramolecular collision and hence dissipation of energy as heat. Irreversible thermal damage to the retina occurs after the ambient temperature is raised by at least 10°C. Depending on the extent of damage, cells may undergo apoptosis, necrosis or immediate cell death [55]. This kind of injury occurs in laser light photocoagulation and optical coherence tomography-guided laser injuries [25]. Photochemical damage is caused by oxygen free radicals. Light in the high-energy portion of the visible spectrum interacts with chromophore molecules like photoreceptors, flavoproteins, heme proteins, melanosomes and lipofuscin present in the retina and retinal pigment epithelium (RPE). These chromophores then release energy to oxygen, generating singlet oxygen species. These free radicals lead to the oxidation of polyunsaturated fatty acids and protein moieties that break down membranous structures. Retinal photoreceptors, especially outer segments, possess large amounts of membranes and are hence more susceptible to free radical-mediated damage [25, 55]. Rapid introduction of energy into melanosomes generates mechanical compressive and tensile forces, causing photomechanical damage. These photomechanical forces result in micro cavitation bubbles, lethal to RPE and other cells [25, 55]. Photo-ablation ensures the damage to photoreceptors, the functional cell types of vision. Photoreceptors when selectively damaged in the retina respond via rod progenitors and Müller glia depending on the extent of damage to the retina.

With the advent of research in transcriptomics, researchers have come up with the association of several genes with retinal disorders. This knowledge has been implied in transgenics and genome editing techniques including TALENS, CRISPR-Cas and morpholino-based gene silencing to mimic the disease in model organisms. Various animal models including fish, amphibians and mammals have been developed to mimic retinal diseases like retinitis pigmentosa, diabetic retinopathy, macular degeneration and glaucoma. Transgenic approaches involving nitroreductase/metronidazole have been used in zebrafish to cause cell-specific ablation (reviewed in [25]). The retinal damage paradigms used in different animal models are given in Table 1. Despite the differences in the mode of injury and the species used, the fundamental principle of regeneration remains similar. In all the cases, there is a necessity to form progenitors from pre-existing cells before the differentiation into various retinal cell types.

## REGENERATION MECHANISMS

**Figure 3:**
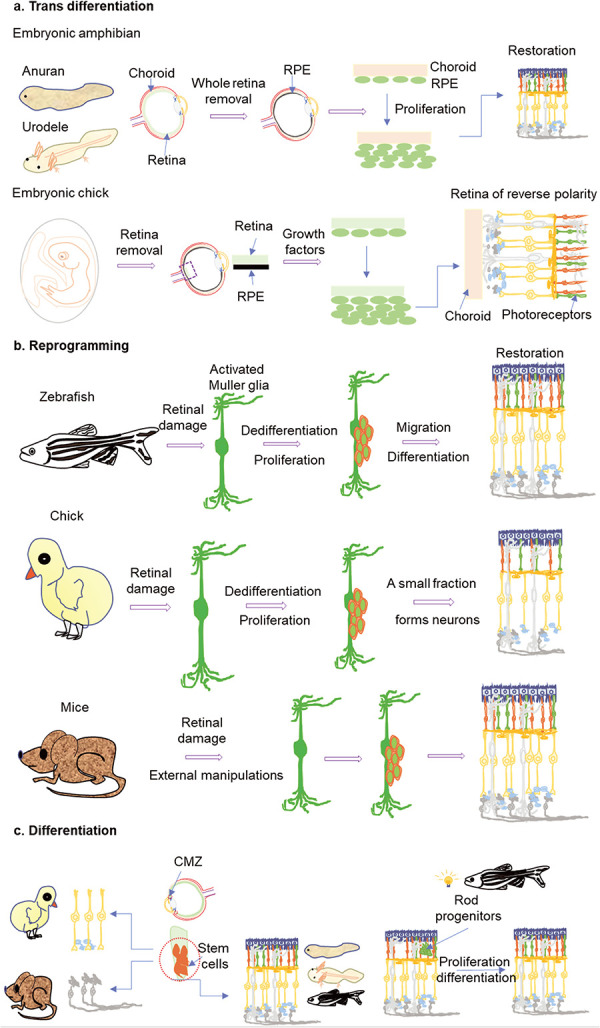
An overview of three different retina regeneration mechanisms followed in the vertebrates. (a) The transdifferentiation of RPE in embryonic stages of amphibians and chicks after whole retina removal. The RPE regenerates the whole retina by losing its pigmentation, changing morphology and proliferating to differentiate into different neuronal retinal cell types. In anuran and urodelian amphibians the RPE needs the presence of a choroid as a support to form the whole retina. In contrast, a portion of neural tissue (retina) ensures retina regeneration in chick embryos. Still, it is of reverse polarity, i.e. the photoreceptor layer faces the vitreous humor, and ganglion cells are toward the vascular layer (choroid). (b) The post-retinal damage reprogramming of Müller glia in zebrafish, chick and mice involves sensing damage by glial cells, which then dedifferentiate to form multipotent stem cells. The dedifferentiated Müller glia divide and migrate to different retinal layers by interkinetic migration, where they differentiate to form lost retinal cell types and restore the vision. The regeneration by Müller glia reprogramming is inherently successful and complete in zebrafish, while in chicks, not all proliferated Müller glia form neuronal cell types. Mice Müller glia are reluctant to reprogram, but in recent studies, the genetic manipulations and/or inhibition of HDACs have enabled their Müller glia to regenerate whole retinal tissue. (c) The CMZ containing constantly proliferating cells at the retinal periphery contributes to retinal growth in adult zebrafish and amphibians throughout their lives. In chick retina, CMZ contributes to neuronal cell types but only amacrine and bipolar cells, up to one-month post-hatching. CMZ is also known to contribute to adult mice retina growth by forming only ganglion cells. In response to light-mediated damage, the rod progenitors, with their origin either in the outer nuclear layer or from progenitors in the inner nuclear layer, contribute to the restoration of lost photoreceptors in the zebrafish retina.

Regeneration relies on replacing damaged or lost cells that happen through different mechanisms, including dedifferentiation, trans-differentiation or reprogramming [43]. Dedifferentiation involves changing terminally differentiated cells to a less differentiated state. These less differentiated cells within their lineage can then divide and replace the lost cells in their vicinity by changing into their final differentiated stage. In trans-differentiation, the already differentiated cells change lineage to form another cell type. To replace the damaged or lost tissue, existing differentiated cells either first dedifferentiate and then differentiate to a new cell lineage or directly change to form new cells. In reprogramming, a fully differentiated cell reverts to its pluripotent stage. In response to injury or damage, fully differentiated cells start dedifferentiation, attaining stem cell-like characteristics which then proliferate and differentiate into different cell types, restoring the lost tissue (Fig. 3). Efficient retina regeneration ensues in cold-blooded vertebrates like fish, urodele amphibians, frogs and embryonic stages of rodents and birds. In these animals, different cellular sources contribute to retina regeneration. These cells, including neural stem cells at the retinal periphery (ciliary marginal zone, CMZ), Müller glia, rod progenitors and RPE, adopt any of the above modes for regeneration to pursue (Table 2).

**Table 2:** The retina regeneration mechanisms adopted at different developmental stages of mammalian and non-mammalian vertebrates enlisting the retinal cell types involved and the status of regeneration in them

Animal	Animal age and retinal progenitors involved	Mechanism	Regeneration status
Fish	**Adult**		
	*CMZ* [60]	Asymmetric division along radial axis	RPE regeneration
	*RPE* [71, 225]	Proliferation and differentiation	Continuous Retinal growth
	*Muller glia* [78, 79, 86]	Reprogramming	Complete and functional retina regeneration RPE regeneration
	**Embryo**		
Amphibians	**Embryonic**		
	[249, 250]	Developmental regrowth	Functional eye regrowth which declines with age
	**Tadpole**		
	*CMZ* [59]	Proliferation and differentiation	Retinal growth at margins
	*Muller glia* [233, 251]	Reprogramming	More efficient in aged animal
Birds	**Embryonic**		
	**Adult**		
	*CMZ* [66]	Proliferation and differentiation	Whole retina regeneration
	*RPE* [69, 70]	Transdifferentiation	Neural retina and lens regeneration
	*Muller glia* [251]	Reprogramming	More efficient in aged animal
Birds	**Embryonic**		
	*RPE* [73, 74, 76]	Transdifferentiation with FGF2 or lin28 and a part of neural retina	Retina of reverse polarity
	**Post-hatch**		
	*CMZ* [252]	Proliferation and differentiation	Amacrine and bipolar cells
	*CMZ* [253]	With growth factors (FGF2 and insulin)	Amacrine, bipolar and ganglion cells
	*Muller glia* [80, 88, 89]	Reprogramming (NMDA, FGF2, CNTF)	
Mammals	**Embryonic**		
	*CMZ* [65]	Proliferation, translocation and differentiation	RGCs generation
	**Post-natal**		
	*Muller glia* [193]	Reprogramming (forced Ascl1 expression)	Amacrine, bipolar and photoreceptor cells
	**Adult**		
	*Muller glia* [37, 42, 89]	Reprogramming (NMDA, FGF, insulin, retinoic acid)	Few bipolar, rod and amacrine cells
	*Muller glia* [90]	Reprogramming (forced Ascl1 expression with HDAC inhibitor)	Functional retina regeneration with more bipolar cells

### Differentiation

Dedifferentiation in the strict sense is not reported during retina regeneration. Here, the consistently growing population of stem cells residing in the CMZ contributes to either retinal growth or regeneration by differentiating into different retinal cell types. In amphibians and teleost fish, the retina grows proportionately to eye or body growth. This continuous growth is due to stem cells residing throughout their life in CMZ. In frogs, most of the retina forms during the tadpole stage from CMZ, with only marginal growth had happened during embryonic development [59]. The stem cells residing in CMZ also contribute to zebrafish retinal growth by dividing asymmetrically in the radial axis and adding concentric rings of new cells [60]. The newly added cells differentiate to form different neural retinal cell types in the proposed sequence from ganglion cells first to cones, horizontal cells, amacrine cells, rods and bipolar cells. After the neurogenesis phase is over, the remaining cells differentiate into Müller glia, which are the last to form and have functions similar to that of astrocytes in the CNS [61]. Chicks’ eyes continue to grow for about a month after hatching with retinal development by cells within the CMZ. However, unlike amphibians and fish, only amacrine and bipolar cells are added to the chick retina under normal physiological conditions without any external factor supplementation. The inability of stem cells in chick CMZ to generate all types of neurons may not be intrinsic to progenitors. Still, it could be due to local factors, which could be overcome by exogenous regulatory molecules like insulin, FGF2, etc. [62, 63]. Retinal stem cells are also identified in adult mice CMZ [64] and shown to form ganglion cells by traveling laterally from CMZ to the neural retina in embryonic stages (E10-15) [65]. CMZ is not the primary source of stem cells after injury in regeneration-competent animals, including fish and amphibia. However, a study in *Xenopus tropicalis* has shown that CMZ participates in complete retina regeneration after total retina removal [51]. Thus, the differentiation of CMZ as a mode of regeneration is often limited in its efficacy except in *Xenopus*.

### Transdifferentiation

The embryonic stages of anuran amphibians and avian embryos can regenerate their retina even after its complete removal, and this regenerative capability is retained even up to adulthood in some urodelian amphibians [67]. In these animals, retina regeneration occurs through RPE transdifferentiation. The RPE restores the damaged retina by dedifferentiating into proliferative neurogenic progenitors [68]. Dedifferentiating RPE loses its pigmentation, changes morphology, detaches from the basement membrane [68] and starts expressing progenitor markers such as Klf4, Sox2, Pax6 and c-Myc [69]. Transdifferentiation requires interaction between the connective tissue and RPE. In the newt, the choroid acts as a connective tissue while in the embryonic chick it is a fragment of the neural retina [67]. Whole retina regeneration is also evident in post-metamorphic *Xenopus laevis* by transdifferentiation of RPE and differentiation of stem cells in CMZ [70]. In adult fish, RPE can regenerate itself after its ablation but does not contribute to retina regeneration [71, 72]. Notably, the avian embryos do not regenerate the retina spontaneously from RPE, but growth factor treatments such as fibroblast growth factor (FGF) [58] or lin28, an RNA binding protein that is important pluripotency inducing factor involved in reprogramming [74], are known to induce RPE transdifferentiation in them. Furthermore, unlike amphibians, embryonic chicks regenerate a nonfunctional retina of reverse polarity (i.e. photoreceptor layer faces the vitreous) and RPE-derived cells do not transform into RPE itself [75, 76]. Though *in vitro* studies have shown FGF2 to transdifferentiate early differentiated embryonic rat RPE to neural retina up to a certain stage [77], mammalian RPE cells *in vivo* are incompetent to reprogram into neural retina. These studies suggest that transdifferentiation as a means of retina regeneration is ineffective in non-amphibian vertebrates.

### Reprogramming

Reprogramming of Müller glia is the potential source of retina regeneration in zebrafish [78, 79], chicks [80] and mammals [37, 42]. Müller glia are the non-neuronal cells with nuclei in the inner nuclear layer and cytoplasmic extensions spanning all the retinal layers. The unique morphology of Müller glia enables them to sense the damage in any of the retinal layers induced by either light, chemicals, cell-specific genetic ablation or mechanical injury [81]. Soon after the tissue damage, the chromatin and transcriptome of Müller glia change to allow them to dedifferentiate [81–83]. This reprogrammed Müller glia enters the cell cycle and starts expressing early neural progenitor markers [61, 84, 85]. In 2006, the use of 1016α1T: GFP transgenic fish, in which adult zebrafish tubulin1α promoter drives GFP expression in injury responsive MGPCs, established Müller glia as the cellular source of fruitful retina regeneration [79]. Since then, several studies have been there to elucidate molecular mechanisms of zebrafish Müller glia reprogramming [9, 61, 81, 86, 87]. The very first evidence of Müller glia as a source of regenerated neuronal cells is from the chick retina [80]. In response to retinal damage, Chick Müller glia enter the proliferative state and express proneural genes [82]. The reprogrammed chick Müller glia are sustained for a long time, expressing undifferentiated cell markers [80, 88], but even after attaining progenitor-like characteristics, the majority of them do not produce neurons probably due to the non-attainment of mature retinal progenitor cell identity [61]. Reprogramming mammalian Müller glia needs either growth factor supplementation, epigenetic modification, transgenic approaches or a combination of them [37, 89, 90]. Without these manipulations, mouse Müller glia does respond to retinal damage, by migrating and expressing progenitor and cell cycle-specific markers but does not enter the cell cycle [91]. The inherent inability of mouse Müller glia to reprogram could be due to epigenetic factors including DNA methylation [92], histone modifications [90] or cell cycle inhibition [91]. In general, the reprogramming of the Müller glia through genetic and epigenetic mechanisms allows the smooth switching into a proliferating population of progenitors, which gives rise to major retinal cell types during retina regeneration.

In the zebrafish retina, continuous growth is supported by stem cells in CMZ and by the continued addition of rod photoreceptors [93]. These rod progenitors arise from a population of slowly dividing cells in the INL. These cells form radially elongated neurogenic clusters and migrate from INL to the ONL, generating rod precursors [94]. Even in the uninjured zebrafish retina, Müller glia proliferates at a low rate expressing the multipotent progenitor marker, Pax6. Müller glia-derived progenitors, which migrate to ONL express Crx (cone-rod homeobox), are the retinal progenitors generating rod photoreceptor lineage [78]. These rod progenitor cells, which originate in INL, divide and migrate to ONL, proliferate further before differentiating and are known to replenish the rod photoreceptors [95]. In the case of photoreceptor-specific ablation, when a retinal injury is chronic or not strong enough to activate Müller glia, the rod progenitors respond to damage [96, 97]. In the diabetic model of zebrafish with *pdx1* homozygous mutation rod and cone cells regenerate from slowly dividing neurod: GFP expressing progenitors with their origin in ONL [98]. The pancreatic and duodenal homeobox 1 (*pdx1*) mutant fish represents diabetic features with reduced beta cells and insulin levels along with elevated glucose levels. In adult zebrafish, the cells destined to become photoreceptors express Neurod [99]. Moreover, the photoreceptors originate without Müller glial activation, which suggests the involvement of rod progenitors in the replenishment of both rod and cone cells [98, 100].

## IS REGENERATION SIMILAR TO EMBRYONIC DEVELOPMENT OR CANCER?

**Figure 4:**
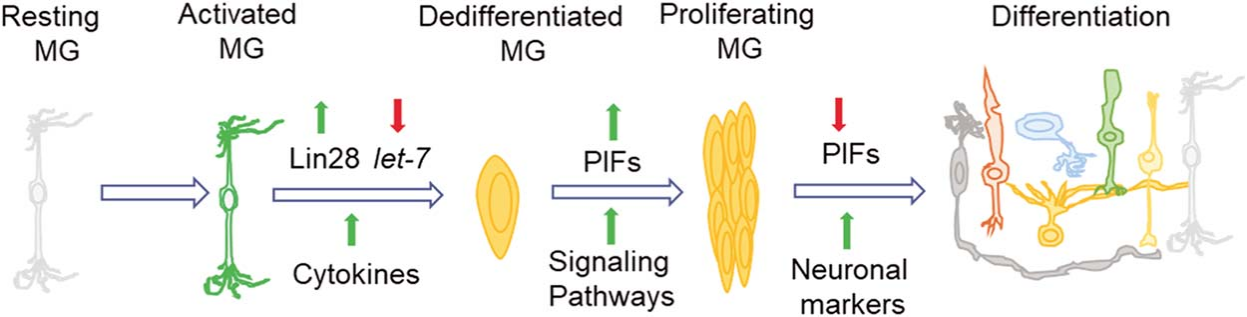
The diagram depicts the set of molecular events leading to Müller glia reprogramming and retina regeneration focusing on zebrafish. Any retinal damage induces the activation of Müller glia (MG) from the resting stage in the intact retina. A number of factors lead to dedifferentiation of activated MG attributed mainly to the increase in Lin28 expression, which degrades *let-7* miRNA. This series of events is accompanied by a hike in pro-inflammatory cytokines and pluripotency inducing factors (PIFs), which lead to activation of many signaling pathways and MG proliferation. A decline follows the proliferative phase in expression levels of PIFs with simultaneously increased expression of neuronal markers culminating in the generation of different retinal cell types with MG cells as the last to form.

Tissue regeneration often shares hallmarks of embryonic development, despite the differences. Several recent studies reveal that various gene regulatory networks active during tissue regeneration are similar to that of embryonic development (reviewed in [101]). During embryonic development, the retina grows as an outgrowth of the forebrain, and different neuronal cell types form from a multipotent progenitor cell. These multipotent progenitors can undergo multiple rounds of cell division, generating different retinal cell types in a rough sequence. A ‘clock’ controls this sequence of generating different cell types during embryonic development with the transition of progenitors from making early cell types to those generated later. A set of gene regulatory events and transcription factors control this transition, driving the acquisition of neural fate and then sequential generation of different cell types. After a retinal injury, this set of events needs to be restarted for restoring different retinal cell types. The Müller glia and RPE, in addition to their immense division capability, also retain the ability to ‘dedifferentiate’ and ‘transdifferentiate’ into progenitor cells. These progenitors resemble those present during embryonic development stages; hence, responding cells seem to follow ‘winding the clock back’ during retina regeneration. In amphibians, the RPE-derived progenitors regenerate the retinal cell types in the same order as during development [102]. Chicks Müller glia proliferate after a retinal injury, but only a few differentiate into neuronal cell types consisting mainly of interneurons [80, 88, 103]. Proliferating Müller glia in chick retina express some progenitor cell markers as Ascl1, Chx10, Pax6, Klf4 and cFos but do not turn on the Otx2, a feature of photoreceptor cells [80, 104, 105]. In zebrafish, after an acute injury, Müller glia-derived progenitors can restore all the retinal cell types. The order of generating new neurons is similar to that of other vertebrates [78, 79]. The early expression of Atoh7 marks the developing ganglion cells as the first cells generated during zebrafish development [106]. During retina regeneration, Müller glia in fish express progenitor-cell associated transcription factors like Pax6, Atoh7, Islet1 and Otx2 [34, 81, 85], and the temporal order of proneural transcription factors is the same as during development [61]. It suggests the ability of fish Müller glia to ‘wind back’ their molecular clock to that of a developmental progenitor state [85]. One of the master regulators behind zebrafish retina regeneration seems to be Lin28a. Soon after the retinal injury, Müller glia starts expressing Lin28, repressing the microRNA (miRNA)-*let-7*. The Lin28-mediated suppression of *let-7* allows Müller glia specific expression of pluripotency factors and efficient retina regeneration (Fig. 4) [107]. The role of Lin28 is conserved across the diverse genera, and it regulates cell fates during development by regulating miRNAs [108–110]. A recent study also shows the transcriptional similarity between the fish Müller glia-derived progenitors and embryonic progenitors [82]. Despite the differences in the cellular niche, the damaged retina often recapitulates various developmental biology events during a regenerative response.

The epithelial to mesenchymal transition (EMT) is an essential cellular phase during cancer. The onset of cancer involves EMT that enables the rapid proliferation and subsequent migration of the cancerous cells to various parts of the affected organism. The opposite mesenchymal to epithelial transition (MET) contributes to the establishment of a new cellular phenotype. Similar steps are needed even during retina regeneration. There are several gene expression events occurring in the zebrafish Müller glia similar to MET, which contribute to the reprogramming and induction of progenitor cells. The cellular reprogramming into pluripotent stem cells is also physiologically related to MET. Several studies on MET during reprogramming have proven that SNAI1 (SNAIL), which is a facilitator of EMT, causes a reduction in reprogramming efficiency both in human and mouse cells [111]. During zebrafish retina regeneration, we see a decline in Snail family genes suggesting the existence of a MET-like situation to induce progenitor cells [112]. Moreover, other findings also support that EMT not only provides cell motility but is also capable of inducing stem-cell properties alongside preventing cellular apoptosis and senescence [113–115]. During zebrafish retina regeneration, the progenitor cell proliferation seen after an injury is dependent on TGF-β signaling that contributes to the downregulation of E-cadherin [112]. The zinc-finger transcription factors ZEB1 and ZEB2 induce EMT by suppressing E-cadherin and hence reorganization of epithelial cells to become migratory mesenchymal cells, a characteristic of cancer metastasis. Zeb1 and Zeb2, are directly controlled by TGF-β signaling during zebrafish retina regeneration leading to the proliferation of the retinal progenitors [112]. Thus, Müller glia reprogramming is initiated with EMT followed by MET before switching into proliferating progenitors that differentiate into retinal cell types. Such observations during retina regeneration draw a parallel with embryonic development and cancer.

## IMMUNE RESPONSE IN RETINA REGENERATION

**Figure 5:**
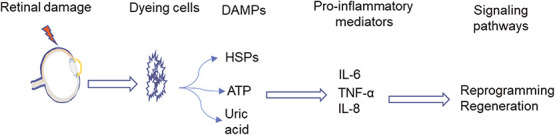
Post-retinal damage induction of immune response leads to successful retina regeneration. Any sort of retinal damage induces cell death as a result of which the dying cells release DAMPs, including heat shock proteins (HSPs), adenosine triphosphate (ATP) and uric acid. These DAMPs then induce the immune cells to release pro-inflammatory cytokines such as IL-6, IL-8 and TNF-α. The cytokine surge culminates in the induction of various signaling pathways resulting in the reprogramming of responding cells and retina regeneration.

After the tissue injury, damaged cells release damage-associated molecular patterns (DAMPs), which are recognized by pattern recognition receptors. DAMPs, including purine metabolites such as ATP or uric acid, heat shock proteins and high mobility group 1 protein, are sequestered intracellularly in intact cells [116]. But upon the damage, injured cells release these DAMPs extracellularly, activating both classical and non-classical pattern recognition receptors, including toll-like receptors, Nod-like receptors, etc. These events lead to the induction of downstream signaling pathways and the production of pro-inflammatory signals (Fig. 5). These signals recruit resident and circulating immune cells to the injury site and promote chemical mediators’ production and release (reviewed in [117]). The innate immune response mediated instant and acute inflammation helps restore tissue homeostasis and activate healing. But dysregulation of inflammation can lead to chronic inflammation, which is harmful to injured tissue and does not culminate in regeneration [118]. In regenerating tissues, inflammatory signals enhance chromatin remodeling for access to DNA by reprogramming factors and promote proliferation in reprogrammed cells [119, 120]. Upon retinal injury, the mammalian Müller glia undergoes reactive gliosis. The reactive gliosis seems to be neuroprotective initially, but it eventually leads to neuronal cell loss and scarring [116]. In zebrafish also, retinal injury induces an inflammatory response, but instead of the glial scar, it pursues Müller glia proliferation and replacement of lost retinal neurons. A recent study with transcriptomic profiling of chick, zebrafish and mice retina suggests mice Müller glia quiescence due to nuclear factor I (*Nfia/b/x*) and *Sox5*. The mice Müller glia express these transcription factors at the resting stage, which are downregulated soon after retinal damage but get upregulated to match the resting Müller glia at later stages [82]. These NFI factors maintain mice Müller glia quiescence, preventing transition to a progenitor-like state and neurogenesis during development as well as post-retinal damage [82, 121]. Injury-induced inflammation also plays a vital role in postnatal chick retina regeneration, where it activates the reprogramming of Müller glia. Still, persistent activation of the NF-κB pathway inhibits the proliferation of progenitors [122]. The complement component C3a alone induces embryonic chick retina regeneration via STAT3 activation and induction of pro-inflammatory cytokines, TNF-α, IL-6 and IL-8 [123].

Microglia are the resident macrophages acting as immune cells in the nervous system, which sense and respond to insult by producing cytokines. After the retinal injury, microglia play a crucial role in modulating Müller glia response, and microglial absence hinders chick or zebrafish Müller glia proliferation [87, 124]. The response of microglia can be pro- or anti-inflammatory depending upon their polarization or the duration up to which they stay active. During zebrafish retina regeneration, suppression of immune response before retinal damage delays, but the post-injury application of immune suppressants accelerates Müller glia/photoreceptor regeneration. The induction of inflammatory response is necessary for zebrafish retina regeneration, but it needs a timely resolution to allow successful progression [125]. The microglia/macrophage-mediated inflammation is a key regulator of mTOR in the Müller glia enabling mTOR-mediated retina regeneration [126]. Pharmacological and genetic ablation reveals the mTOR pathway as an integral part of zebrafish RPE regeneration also, where it recruits macrophages/microglia to the injury site that in turn further activates the mTOR pathway [127]. The microglia are also crucial for zebrafish RPE regeneration, where RPE expresses cytokines including interleukin34 (*il34*) as a leukocyte recruitment factor [128]. In contrast to this, the microglia in the mouse retina are known to induce inflammatory genes reducing neurogenesis [129]. The knowledge of the immune response during retina regeneration in various vertebrates reveals a crucial involvement of various immunological molecules that govern a smooth regenerative response.

## MOLECULAR BASIS OF RETINA REGENERATION

The retinal injury induces Müller glia and efficient retina regeneration in cold-blooded animals like teleost fish but not so in mammals. Different research groups in varying parts of the world have uncovered the molecular mechanisms underlying the regenerative capability of vertebrates. Several developmental signaling and other pathways are known to induce Müller glia reprogramming or maintain their quiescent state (Figs 4 and 5).

### Cytokines

Retinal damage causes cell death and the release of pro-inflammatory cytokines. In the *Xenopus* eye, retinal removal induces Il-1β and Tnf-α, which upregulate *mmp9* and *mmp18* essential for RPE transdifferentiation [134]. In the photoreceptor regeneration using transgenic zebrafish, TNF-α accounts for the transition of Müller glia from non-proliferative gliosis to a regenerative state [131]. Upon retina damage, dying retinal neurons produce Tnf-α, which is necessary for Müller glia proliferation in zebrafish retina regeneration [135]. After neuronal damage, the dying Müller glia release cytokines and inflammatory Mmp9. Mmp9 is a matrix metalloproteinase, which plays a crucial role in inflammation by cleaving precursor or mature cytokines, making them active or rendering them inactive. Mmp9 plays an essential role in resolving an inflammatory response, as its mutants express higher levels of pro-inflammatory cytokines such as TNF-α [132]. The importance of Mmp9 is also supported by the induction of *mmp9* soon after damage and its strong upregulation in MGPCs [133]. During photoreceptor regeneration, Mmp9 plays a vital role in cone photoreceptors’ survival [132]. In zebrafish, the photoreceptor damage upregulates *mmp9* in dividing Müller glia and photoreceptor progenitors. The expression of *mmp9* is also induced by TNF-α, even in intact retinae. Insulin, IGF-1, HB-EGF and cytokines synergize to stimulate Müller glia reprogramming even in the uninjured zebrafish retina through a set of signaling pathways [136]. The leptin and Il-6 family cytokines promote zebrafish retina regeneration post-injury by Jak/Stat signaling [137]. The midkine cytokine is necessary for G1 to S phase transition in zebrafish. Its loss results in reactive gliosis of Müller glia, which may be one of the reasons for unsuccessful mammalian retina regeneration [130]. While the persistent release of cytokines is regulated through various cell signaling events, their necessity is proven to be a hallmark of retina regeneration across multiple model systems.

### TGF-β signaling

TGF-β signaling governs many biological processes that have an anti-proliferative role in various biological systems. In zebrafish, TGF-β3 promote retina regeneration via a canonical pathway involving *mycb* and *junb* gene family activation [138], while other study reports its inhibitory role probably via a non-canonical path [139]. We have found the biphasic role of the TGF-β1 signaling pathway during zebrafish retina regeneration, where it regulates regeneration-associated genes and miRNAs for successful Müller glia reprogramming, proliferation and then differentiation to maintain the retinal homeostasis [112]. TGF-β signaling plays a neuronal cell death protective role during mice’s retina development [140], while in adult mice, its absence in microglia leads to neuroinflammation and retinal degeneration [141]. Post-retinal damage, TGFβ/Notch signaling axis, reprograms mouse Müller glia to epithelial lineage and glial scar formation [142]. TGF-β1 and TGF-β2 activate non-canonical p38MAPK signaling in mice leading to gliosis. It is interesting to note that the anti-proliferative TGF-β signaling in mammals, which often turns pro-proliferative during the cancerous conditions, is pro-proliferative during zebrafish retina regeneration.

### Pluripotency factors

After the initial reprogramming events, Müller glia attain stem cell-like characteristics with induction of pluripotency factors [107]. We have explored the role of *oct4* during zebrafish retina regeneration, where initial pan-retinal expression of *oct4* regulates Müller glia reprogramming via regulation of regeneration-associated genes, E-cadherin and micro-RNAs. Oct4 also plays an essential role in cell cycle exit by suppressing pro-proliferative genes in collaboration with Hdac1 [143]. The morpholino-mediated knockdown of *sox2*, *klf4* and *nanog*, too, impair the zebrafish retina regeneration (unpublished data). We have shown Myca/b to be an early induced pluripotency factor that regulates zebrafish Müller glia reprogramming via activation of *ascl1a* and *lin28* [144]. The Sox2 is a crucial player in zebrafish Müller glia reprogramming and proliferation, where it activates *ascl1a* and *lin28a*, and its loss affects cone photoreceptor regeneration [145]. In medaka fish, Sox2 and other factors maintain Rx2 expression in CMZ, regulating stem cell fate toward neural retina or retina pigment epithelium [146]; alongside this post-retinal damage, sustained Sox2 expression allows a regenerative response similar to zebrafish [147]. In chicks, Sox2 reprograms RPE differentiation toward retinal neurons [148]. The conditional expression of Ascl1 and Lin28 in uninjured zebrafish and mice retina stimulates sparse Müller glia proliferation. The simultaneous inhibition of Notch signaling enhances neuronal regeneration in the former but not in the latter [149]. In rats, subretinal injections of OCT4 reprogrammed human pluripotent stem cells cause RPE generation [150]. The spontaneous induction of pluripotency-inducing factors in the injured retina or RPE paves the way for switching into a proliferating population of progenitors during retina regeneration.

### mTOR pathway

Retinal damage induces the release and production of various factors, activating several signaling pathways. Stab wound injury in zebrafish induces mTOR signaling and is essential for Müller glia dedifferentiation and proliferation forming MGPCs by activating regeneration-associated factors like *lin28a*, *ascl1a*, cytokines and cell-cycle regulators [126]. Further, the downregulation of the tumor suppressor Pten that activates the Akt–mTOR pathway in the zebrafish retina is also pivotal in Müller glia reprogramming [152]. Retinal damage triggers mTOR signaling in activated Müller glia and its inhibition impairs MGPCs proliferation even in EGF2 treated retinae. Inhibition of the mTOR pathway also surpassed the MGPC-promoting effects of glucocorticoid, sonic hedgehog (Shh) and wnt signaling in chick retina [151]. These observations support the view that the mTOR pathway is important for successful retina regeneration.

### Hippo signaling

Hippo signaling is an important developmental pathway regulating eye growth through YAP and TAZ. Yap is required for the Müller glia to respond to an injury by regulating their cell cycle re-entry and progenitor cell formation, leading to the differentiation of new photoreceptors in zebrafish [156]. Yap suppresses photoreceptor differentiation during zebrafish development by suppressing Rx1-mediated activation of photoreceptor genes [157]. Knockdown of *yap1* impairs Müller glia proliferation and neurogenesis after photoablation in the zebrafish retina [82]. Upon retinal damage, YAP gets upregulated in mice and *Xenopus* Müller glia, and its conditional deletion inhibits cell-cycle gene expression and hence reactive gliosis in the former [154]. In postnatal mice, TAZ overexpression compensates for the loss of YAP. In adult mice-retina, YAP expression pertains to Müller glia. Its loss is not compensable by TAZ, leading to cone degeneration and Müller glia dysfunction in aged animals [153]. The hippo pathway in ‘on-state’ inhibits YAP nuclear translocation and blocks mammalian retina regeneration [155]. Müller glia-specific deletion of its component genes and transgenic overexpression of hippo unresponsive YAP leads to Müller glia reprogramming to a progenitor-like state [155]. In mice, the YAP-EGFR axis plays an essential role in the exit of Müller glia quiescence and proliferation in response to injury [154]. The YAP overactivation in the mouse Müller glia induces their reprogramming into highly proliferative cells, a feature necessary in all retinal regeneration events [154]. The developmental pathway of Hippo signaling in its ‘off-state’ is crucial to the retina regeneration in different vertebrates.

### Shh signaling

Shh is another developmental pathway whose dysregulation is known to cause anomalies such as cyclopia. The Shh pathway is very important in several species studied in the retina regeneration context. In the developing retina of zebrafish and *Xenopus*, the Hedgehog pathway shortens the G1 and G2 phases, thus speeding up and early exit of the cell cycle [158]. Early Shh activation induces gliosis, proliferation and neuroprotection and its continued activation facilitates amacrine and ganglion cell differentiation in injured zebrafish retina [159]. In zebrafish, the blockade of Shh signaling completely abolishes the regenerative potential, and this pathway activates regeneration-associated genes to facilitate zebrafish retina regeneration [133]. Overexpression of recombinant Shh also activates proneural gene Ascl1 in both zebrafish and mice [133]. In chick retina, Shh overexpression alone induces proliferation from the anterior margin of the eye without external FGF2 [160]. Activation of the Shh pathway with its agonist purmorphamine induces transdifferentiation of Müller glia into rod photoreceptors in rat retina [161]. Shh also protects ganglion cells from chronic hypertension in adult rats [162]. Taken together, these observations suggest the crucial roles of yet another developmental pathway, Shh signaling, during retina regeneration.

### Wnt/beta-catenin signaling

Wnt signaling is essential in embryonic development, stem cell maintenance, tissue repair and cancer [163]. During zebrafish retina regeneration, Wnt/β-catenin signaling plays a crucial role, where inhibition of GSK-3β to stabilize the β-catenin alone induces Müller glia dedifferentiation and retinal cell types formation even in the uninjured retina [164]. After retinal damage, Wnt signaling is stimulated via an early pan-retinal induction of Insm1a that suppresses pan-retinal *dkk* (a negative regulator of wnt signaling), *ascl1a*, and its expression to restrict Ascl1a-Insm1a-Dkk axis back to the site of injury until the regeneration is completed [165]. Wnt and BMP signaling reprogram the neural retina into RPE in the chick retina [166]. Activation of Wnt/β-catenin signaling promotes the reprogramming of Müller glia to precursor state, proliferation and neural regeneration in mice [167, 168] via the Lin28/let-7 pathway even in the uninjured retina [169]. Mice Müller glia after β-catenin gene transfer also reprograms to form rod photoreceptors on rod specific gene transfer [170]. These observations emphasize the pivotal roles played by Wnt signaling in many vertebrate models of retina regeneration.

### MAP/ERK pathway

The MAP/ERK pathway is very important for cells to respond to external signals and subsequent cell proliferation. The MAP/ERK pathway is essential in many biological events from developmental biology, cell division, tissue repair and cancer. Several external signals, including EGF2, IGF-1, BDNF, cytokines, neurotrophins and DNA damage, activate the MAPK pathway. During zebrafish retina regeneration, ERK signaling gets activated in Müller glia by induction of multiple growth factors, including HB-EGF, IGF1, FGF2 and insulin. This pathway then acts synergistically with other signaling pathways to promote transdifferentiation and Müller glia reprogramming (reviewed in [171]). Soon after a retinectomy, ERK signaling in the adult newt gets activated with p-ERK nuclear translocation and loose cell contact, causing β-catenin nuclear translocation. Extracellular FGF2 keeps ERK activated, which along with β-catenin and HB-FGF signaling, leads to cell-cycle re-entry, transdifferentiation and proliferation of RPE. EGF2 administration in *X. laevis* and chicks activate the ERK pathway to induce *pax6* expression and hence RPE reprogramming (reviewed in [171]). In the injured chick retina, the Müller glia showed accumulation of p-ERK1/2, and p-CREB proteins, along with transient expression of cFos and Egr1, an indication of active MAPK-signaling [172]. In mice retina, the p38 MAPK pathway provides LIF (leukemia inhibitory factor) dependent neuroprotection after light-induced degeneration [173]. Altogether, the MAP/ERK pathway is one of the important signaling events soon after retinal damage observed in both mammals and non-mammalian animals.

### Glucocorticoid receptor signaling

The glucocorticoid receptor signaling (GCR) is connected with anti-inflammatory responses, and its agonists are used to treat inflammatory eye diseases. In the zebrafish retina, activation of the GCR pathway before rod photoreceptor ablation delays Müller glia proliferation and replacement of lost rod cells. Its activation after damage enhanced the photoreceptor regeneration, probably by rapid immune response resolution [125]. Intact chick eyes have GCR expression in CMZ, and upon acute retinal injury, it gets upregulated in Müller glia. The activation of the GCR pathway is anti-proliferative by inhibiting the FGF2/MAPK pathway, while its inhibition, in the injured chick retina, promotes Müller glia proliferation and neuronal differentiation [174]. In mice retina, activation of the GCR pathway prevents light-induced photoreceptor death [175] and induces retinal stem cell proliferation in CMZ [176]. These observations suggest the potential of GCR in adapting to different cell physiology and is an essential contributory pathway during retina regeneration.

### Notch signaling

Notch signaling is known to maintain Müller glia quiescence in the uninjured retina. After retinal damage, Notch signaling keeps the injury-responsive cells at the site of injury and regulates the threshold and proliferation of injury-responsive Müller glia [177]. In the undamaged zebrafish retina, inhibition of Notch signaling along with TNFα activation reprograms Müller glia generating different retinal neurons similar to regenerating retina [178]. Inhibition of Notch signaling and forced expression of Ascl1a and Lin28a stimulate widespread multipotent Müller glia progenitors in intact zebrafish retina but not in mice [149]. Notch signaling along with Tgfb3 inhibits zebrafish retina regeneration [139]. Similarly, Fgf8 mediated suppression of Notch signaling stimulates Müller glia proliferation in the young fish retina, while increasing Notch signaling and suppressing Müller glia proliferation in the older fish [179]. Notch signaling mediates the early steps of RPE transdifferentiation in the newt retina, where Notch-1 expressing cells first appear in the regenerating retina [180]. In the chick retina persistently active Notch signaling is inhibitory to neuronal regeneration, while initial activation is essential for Müller glia dedifferentiation; for efficient neuronal cell differentiation, it needs to be suppressed at later time points [103]. LIM/homeobox transcription factor (*Lhx2*, a transcriptional activator) mediated regulation of Notch signaling is essential for Müller glia specification and differentiation in mice retina [181]. In the rat model of retinal degeneration, intravitreal injection of olfactory ensheathing cells preserves the visual function by suppressing Notch signaling and inhibiting Müller glia activation associated with gliosis [182]. These diverse observations of various vertebrate models suggest that Notch signaling plays a unique and stage-dependent role during retina regeneration.

### Jak/Stat and other pathways

The Jak/Stat pathway and its stringent regulation are crucial during embryonic development and diseases such as cancer. Soon after retinal injury in zebrafish, damage-induced cytokines activate the Jak/Stat signaling culminating in Müller glia reprogramming by Ascl1a regulation and successful neuronal regeneration [137]. The NMDA-induced retina damage or growth factor administration activates Jak/Stat signaling in chick Müller glia facilitating proliferation but inhibiting neural differentiation [88]. In mice retina, TNFα activates Müller glia proliferation and inflammatory response instead of neural regeneration via Jak/Stat and MAPK pathways [183]. The inability of mice Müller glia neural regeneration is due to STAT directed binding of Ascl1 to inappropriate targets like Id1 and Id3, which keeps the cells in a progenitor-like state and hence prevents differentiation [184]. Thus, the stringent regulation of STAT and subsequent gene activations are essential for the normal and effective regenerative response in the damaged retina.

Several studies demonstrate that many developmentally essential pathways are active and necessary for efficient retina regeneration. It is tempting to speculate that retina regeneration employs several developmental biology pathways because of the recapitulation of embryonic development during retina regeneration. It is also important to note that several of these pathways contribute to the onset and progression of cancer, which also parallels retina regeneration. However, it is essential to note that despite many developmental and cancer biology pathways being turned on during retina regeneration, the tissue efficiently controls the cell proliferation through apoptosis and avoids shifting into cancerous conditions in normally regenerating organisms such as fishes and frogs.

## EPIGENETIC ASPECTS OF RETINA REGENERATION

Retina regeneration involves responding cells transition from a fully differentiated quiescent state to stem cell-like features. Many genetic and epigenetic events happen during reprogramming, allowing chromatin accessibility and gene transcription [101]. The epigenetic regulations involving DNA methylation, histone modifications and non-coding RNAs enable the switching of differentiated cells to pluripotent-like states. The changes in genes encoding chromatin-modifying proteins are associated with various congenital retinal malformations (reviewed in [185]). DNA demethylation contributes to Müller glia reprogramming in zebrafish, but consistent hypomethylation affects progenitor cell proliferation, migration and differentiation. The changes in methylation patterns were revealed by reduced representation bisulfite sequencing. Indicating that active DNA demethylation occurs soon after damage during zebrafish retina regeneration, allowing Müller glia reprogramming, and then methylation patterns are regained to enable migration and differentiation [83]. The RPE of the chick (embryonic stages 23–25) retina undergoes dynamic changes in bivalent histone marks (H3K27me3/H3K4me3) and DNA demethylation during reprogramming. In the chick retina, tet methylcytosine dioxygenase (TET3) facilitates DNA demethylation and RPE reprogramming, even in the absence of external growth factors [186].

Moreover, promoters of regeneration-associated genes were already hypomethylated in the resting Müller glia of the zebrafish retina, which stayed the same in the progenitor cells [83]. In contrast to zebrafish, the Müller glia in medaka is less responsive to injury forming only photoreceptors. After retinal damage, Sox2 gets downregulated (silenced) in Medaka fish, and upon restoration of Sox2, medaka Müller glia can behave like that of zebrafish [145, 147]. Like the zebrafish retina, the epigenome of mice Müller glia resembles that of late retinal progenitor cells with hypomethylation of regeneration-associated genes [187]. Though mice Müller glia transcriptome changes post damage, it fails to stimulate cell cycle and retinogenic factors to the states observed in early retinal progenitor cells [188]. Soon after the injury, the promoter of *Oct4* is hypomethylated but then returns to methylated status as that of quiescent Müller glia with concomitant changes in DNA methyltransferase (dnmt3b) levels [92]. Since Oct4 plays a vital role during zebrafish retina regeneration, silencing of *Oct4* after mice retinal damage may account for unsuccessful renewal in the latter [143].

Chromatin modifying enzymes are emerging as important regulators of zebrafish retina regeneration. Histone methyltransferases (Dotl1, an H3K79 methyltransferase) and histone deacetylases (Hdac1, histone deacetylase 1) are essential for Müller glia dedifferentiation and proliferation in zebrafish [144, 189, 190]. The mammalian RPE reprogramming inefficiency is also due to the highly methylated promoters of photoreceptor-related genes and repressive chromatin marks on the non-photoreceptor neuronal genes [191]. The *in vitro* studies with mice Müller glia in dissociated cultures demonstrated reactivation of many genes and neuronal generation after Ascl1a overexpression. Chromatin immunoprecipitation analysis revealed a reduction in repressive (H3K27me3) while enhancement in activating (H3K27ac) histone marks at the reactivated genes [192]. The Ascl1a transgenic mice could reprogram their Müller glia, generating neurons (bipolar and amacrine cells) in the early stages (2 weeks old) but not in mature animals [193]. The DNase-seq analysis in adult mice revealed the loss of open chromatin near neuronal genes, and inhibition of histone deacetylases (HDACs), along with Ascl1a overexpression, could make them regenerate successfully [90]. Epigenome modifications are key to the regenerative response in an injured retina both for induction of progenitors and differentiation to various retinal cell types.

### The roles of non-coding RNAs

The non-coding RNAs, including short non-coding RNAs (microRNAs), long non-coding RNAs and circular RNA, play an essential role in several cellular processes. They regulate cell proliferation, differentiation and apoptosis and are also significant players in retinal development and the initiation and progression of retinal diseases (reviewed in [194, 195]). miRNAs are small non-coding RNAs that regulate many biological processes by interfering with the translation of target proteins. The miRNA transcripts need processing by two RNAse III proteins, Drosha and Dicer [196]. Alterations in miRNA expression are associated with retinal diseases such as age-related macular degeneration and glaucoma. The importance of miRNAs during retinal development and regeneration was noticeable after dicer inactivation (reviewed in [197]). Dicer mutant zebrafish and *X. laevis* have defective retinal growth, including cell differentiation and retinal lamination [198, 199]. Though dicer deficient mice did not have evident defects at an early postnatal time, aged mice showed defects in light responsiveness and retinal structure [200] (reviewed in [197]). miRNAs are also essential during retina regeneration as Dicer knockdown after retinal damage impaired proliferation of zebrafish Müller glia [201]. The small RNA sequencing at different time points after light damage revealed some miRNAs to be upregulated (*miR-142b*, *miR-146a*, *miR-7a*, *miR-27c* and *miR-31*). The morpholino-mediated knockdown of these miRNAs proved they are essential for retina regeneration [201]. The levels of some miRNAs get downregulated post-retinal injury, suggesting their involvement in maintaining a quiescent state or inhibiting Müller glia reprogramming. A well-studied miRNA *let-7* maintains Müller glia quiescence, which must get downregulated by Lin-28 for efficient retina regeneration [107, 133, 189]. Similar to *let-7*, the other two miRNAs must be downregulated post-retinal damage, i.e. *miR-203* and *miR-216a*, to allow successful zebrafish retina regeneration. The *miR-203* targets Pax6b, inhibiting the proliferation of progenitors, and *miR-216a* targets Dot1l, inhibiting dedifferentiation and proliferation of Müller glia [190, 202]. The role of other miRNAs, *miR-143*, *miR-145*, *miR-200a* and *miR-200b*, is known during zebrafish retina regeneration. Oct4 and TGF-β signaling regulate these miRNAs to culminate in the efficient and controlled retina regeneration [112, 143]. The manipulation of miRNAs also promotes mammalian retina regeneration *in vitro* (reviewed in [197]). These studies emphasize the importance and necessity of miRNA-mediated gene regulations for efficient retina regeneration.

## ARE WE NEARING MAMMALIAN RETINA REGENERATION?

The increasing number of shreds of evidence from non-mammalian vertebrates suggests that retina regeneration is carried out through efficient orchestration of gene induction and repression in a stringent spatial and temporal fashion. In mammals, soon after injury, Ascl1 [193] and Oct4 [92] are induced similar to that found in the injured zebrafish retina [107, 143]. Several mammalian models also suggest the propensity of retinal regeneration akin to the non-mammalian models in a controlled scenario such as overexpression of Ascl1 [192]. However, despite the inefficiency of regeneration, this type of forced expression of certain master regulators, making the mammalian retina more inclined toward retinal regeneration, suggests epigenetic factors’ possible involvement. Further, the overexpression of Ascl1 along with repression of HDAC [90] or inhibitor of STAT-signaling [184] made the mammalian retina more congenial for regeneration. Apart from the epigenome-influencing factors, various micro RNAs such as miR-25, let-7 and miR-124 also play important roles in regenerative potential in the mammalian retina [203]. Unlike the fishes and amphibians, most other vertebrate models of retina regeneration have a terrestrial life where wound healing is often seen after an injury compared to functional restoration through regeneration. The prevalence of wound healing in land animals compared to their aquatic counterparts is not fully understood. However, it is believed that land animals perform a faster wound healing instead of slow regeneration, probably to avoid the risk of infection or the ability to lead a normal life despite having a compromised organ structure. It is also interesting to note that all vertebrates possess excellent regenerative capability during their embryonic stages where a complete or semi-aquatic environment is present. This ability to regenerate early during development could also be attributed to the less repressive epigenetic environment facilitating gene expression. Even in axolotls, after a forced metamorphosis, which now leads to a terrestrial habitat lacking the robust regenerative capability it had during its aquatic life [204]. The reduced regenerative potential in salamander after the metamorphosis could also be implied to the immunological barrier to regeneration [205]. It would be exciting to explore the regenerative potential of several obligate aquatic mammals. Taken together, it is tempting to speculate that mammals do possess the ability to regenerate as found in non-mammalian vertebrates provided ample opportunity in their genetic and epigenetic landscape.

## FUNDING

P.S. acknowledges her support from the SERB NPDF (PDF/2019/001148) for postdoctoral fellowship. R.R. also acknowledges research funding from Science Education and Research Board SERB, DST, India (EMR/2017/001816), DBT India (BT/PR17912/MED/31/336/2016), STAR grant from DoE (STARS1/180) and support from IISER Mohali.

## CONFLICT OF INTEREST

None declared.[Supplementary-material sup1]

## Supplementary Material

suppl_data_kvac012

## References

[1] Barker N. Adult intestinal stem cells: critical drivers of epithelial homeostasis and regeneration. Nat Rev Mol Cell Biol. 2014;15:19–33 24326621 10.1038/nrm3721

[2] Hogan BL, Barkauskas CE, Chapman HA et al. Repair and regeneration of the respiratory system: complexity, plasticity, and mechanisms of lung stem cell function. Cell Stem Cell. 2014;15:123–38 25105578 10.1016/j.stem.2014.07.012 PMC4212493

[3] Kotton DN, Morrisey EE. Lung regeneration: mechanisms, applications and emerging stem cell populations. Nat Med. 2014;20:822–32 25100528 10.1038/nm.3642 PMC4229034

[4] Yang HC, Liu SJ, Fogo AB. Kidney regeneration in mammals. Nephron Exp Nephrol. 2014;126:50–3 24854640 10.1159/000360661 PMC4337834

[5] Alysandratos KD, Herriges MJ, Kotton DN. Epithelial stem and progenitor cells in lung repair and regeneration. Annu Rev Physiol. 2021;83:529–50 33074772 10.1146/annurev-physiol-041520-092904 PMC9068227

[6] Gemberling M, Bailey TJ, Hyde DR et al. The zebrafish as a model for complex tissue regeneration. Trends Genet. 2013;29:611–20 23927865 10.1016/j.tig.2013.07.003 PMC3812420

[7] Godwin J. The promise of perfect adult tissue repair and regeneration in mammals: learning from regenerative amphibians and fish. BioEssays. 2014;36:861–71 25043537 10.1002/bies.201300144

[8] Wan J, Goldman D. Retina regeneration in zebrafish. Curr Opin Genet Dev. 2016;40:41–7 27281280 10.1016/j.gde.2016.05.009 PMC5135611

[9] Lahne M, Nagashima M, Hyde DR et al. Reprogramming Müller glia to regenerate retinal neurons. Annu Rev Vis Sci. 2020;6:171–93 32343929 10.1146/annurev-vision-121219-081808 PMC8384111

[10] Baden T, Euler T, Berens P. Understanding the retinal basis of vision across species. Nat Rev Neurosci. 2020;21:5–20 31780820 10.1038/s41583-019-0242-1

[11] Hoon M, Okawa H, Della Santina L et al. Functional architecture of the retina: development and disease. Prog Retin Eye Res. 2014;42:44–84 24984227 10.1016/j.preteyeres.2014.06.003 PMC4134977

[12] Rieke F. Mechanisms of single-photon detection in rod photoreceptors. Methods Enzymol. 2000;316:186–202 10800676 10.1016/s0076-6879(00)16724-2

[13] Sampath AP, Rieke F. Selective transmission of single photon responses by saturation at the rod-to-rod bipolar synapse. Neuron. 2004;41:431–43 14766181 10.1016/s0896-6273(04)00005-4

[14] Baden T, Nikolaev A, Esposti F et al. A synaptic mechanism for temporal filtering of visual signals. PLoS Biol. 2014;12:e1001972 25333637 10.1371/journal.pbio.1001972 PMC4205119

[15] Tsukamoto Y, Morigiwa K, Ueda M et al. Microcircuits for night vision in mouse retina. J Neurosci. 2001;21:8616–23 11606649 10.1523/JNEUROSCI.21-21-08616.2001 PMC6762784

[16] Chapot CA, Euler T, Schubert T. How do horizontal cells ‘talk’ to cone photoreceptors? Different levels of complexity at the cone-horizontal cell synapse. J Physiol. 2017;595:5495–506 28378516 10.1113/JP274177 PMC5556172

[17] Thoreson WB, Mangel SC. Lateral interactions in the outer retina. Prog Retin Eye Res. 2012;31:407–41 22580106 10.1016/j.preteyeres.2012.04.003 PMC3401171

[18] Euler T, Haverkamp S, Schubert T et al. Retinal bipolar cells: elementary building blocks of vision. Nat Rev Neurosci. 2014;15:507–19 25158357 10.1038/nrn3783

[19] Baccus SA. Timing and computation in inner retinal circuitry. Annu Rev Physiol. 2007;69:271–90 17059359 10.1146/annurev.physiol.69.120205.124451

[20] Diamond JS. Inhibitory interneurons in the retina: types, circuitry, and function. Annu Rev Vis Sci. 2017;3:1–24 28617659 10.1146/annurev-vision-102016-061345

[21] Franke K, Baden T. General features of inhibition in the inner retina. J Physiol. 2017;595:5507–15 28332227 10.1113/JP273648 PMC5556161

[22] Masland RH. The tasks of amacrine cells. Vis Neurosci. 2012;29:3–9 22416289 10.1017/s0952523811000344 PMC3652807

[23] Sanes JR, Masland RH. The types of retinal ganglion cells: current status and implications for neuronal classification. Annu Rev Neurosci. 2015;38:221–46 25897874 10.1146/annurev-neuro-071714-034120

[24] Dhande OS, Huberman AD. Retinal ganglion cell maps in the brain: implications for visual processing. Curr Opin Neurobiol. 2014;24:133–42 24492089 10.1016/j.conb.2013.08.006 PMC4086677

[25] Sherpa RD, Hui SP. An insight on established retinal injury mechanisms and prevalent retinal stem cell activation pathways in vertebrate models. Animal Model Exp Med. 2021;4:189–203 34557646 10.1002/ame2.12177 PMC8446703

[26] Sharma P, Ramachandran R. Retina injury and retina tissue preparation to study regeneration in zebrafish. Bio Protoc. 2019;9:e3466 10.21769/BioProtoc.3466 PMC7854005 33654957

[27] Matsumoto H, Miller JW, Vavvas DG. Retinal detachment model in rodents by subretinal injection of sodium hyaluronate. J Vis Exp. 2013;79:50660 10.3791/50660 PMC3864357 24056325

[28] Tucker B, Klassen H, Yang L et al. Elevated MMP expression in the MRL mouse retina creates a permissive environment for retinal regeneration. Invest Ophthalmol Vis Sci. 2008;49:1686–95 18385092 10.1167/iovs.07-1058 PMC2613950

[29] Cameron DA, Easter SS Jr. Cone photoreceptor regeneration in adult fish retina: phenotypic determination and mosaic pattern formation. J Neurosci. 1995;15:2255–71 7891164 10.1523/JNEUROSCI.15-03-02255.1995 PMC6578115

[30] Hitchcock PF, Myhr KJL, Easter SS et al. Local regeneration in the retina of the goldfish. J Neurobiol. 1992;23:187–203 1527527 10.1002/neu.480230209

[31] Murphy MJ, Crewther SG. Ouabain inhibition of Na/K-ATPase across the retina prevents signed refractive compensation to lens-induced defocus, but not default ocular growth in young chicks. F1000Res. 2013;2:97 25506418 10.12688/f1000research.2-97.v1 PMC4257142

[32] Maier W, Wolburg H. Regeneration of the goldfish retina after exposure to different doses of ouabain. Cell Tissue Res. 1979;202:99–118 509506 10.1007/BF00239223

[33] Sun C, Mitchell DM, Stenkamp DL. Isolation of photoreceptors from mature, developing, and regenerated zebrafish retinas, and of microglia/macrophages from regenerating zebrafish retinas. Exp Eye Res. 2018;177:130–44 30096325 10.1016/j.exer.2018.08.002 PMC6261699

[34] Sherpa T, Fimbel SM, Mallory DE et al. Ganglion cell regeneration following whole-retina destruction in zebrafish. Dev Neurobiol. 2008;68:166–81 18000816 10.1002/dneu.20568 PMC2581885

[35] Fimbel SM, Montgomery JE, Burket CT et al. Regeneration of inner retinal neurons after intravitreal injection of ouabain in zebrafish. J Neurosci. 2007;27:1712–24 17301179 10.1523/JNEUROSCI.5317-06.2007 PMC6673754

[36] Li V, Wang YT. Molecular mechanisms of NMDA receptor-mediated excitotoxicity: implications for neuroprotective therapeutics for stroke. Neural Regen Res. 2016;11:1752–3 28123410 10.4103/1673-5374.194713 PMC5204222

[37] Karl MO, Hayes S, Nelson BR et al. Stimulation of neural regeneration in the mouse retina. Proc Natl Acad Sci USA. 2008;105:19508–13 19033471 10.1073/pnas.0807453105 PMC2614791

[38] Bai N, Aida T, Yanagisawa M et al. NMDA receptor subunits have different roles in NMDA-induced neurotoxicity in the retina. Mol Brain. 2013;6:34 23902942 10.1186/1756-6606-6-34 PMC3733768

[39] Nelson R, Bender AM, Connaughton VP. Stimulation of sodium pump restores membrane potential to neurons excited by glutamate in zebrafish distal retina. J Physiol. 2003;549:787–800 12730339 10.1113/jphysiol.2003.042051 PMC2342992

[40] Nomura-Komoike K, Saitoh F, Komoike Y et al. DNA damage response in proliferating Müller glia in the mammalian retina. Invest Ophthalmol Vis Sci. 2016;57:1169–82 26975029 10.1167/iovs.15-18101

[41] Sheng W, Lu Y, Mei F et al. Effect of resveratrol on sirtuins, OPA1, and Fis1 expression in adult zebrafish retina. Invest Ophthalmol Vis Sci. 2018;59:4542–51 30208422 10.1167/iovs.18-24539

[42] Ooto S, Akagi T, Kageyama R et al. Potential for neural regeneration after neurotoxic injury in the adult mammalian retina. Proc Natl Acad Sci USA. 2004;101:13654–9 15353594 10.1073/pnas.0402129101 PMC518808

[43] Zhang DQ, Zhou TR, McMahon DG. Functional heterogeneity of retinal dopaminergic neurons underlying their multiple roles in vision. J Neurosci. 2007;27:692–9 17234601 10.1523/JNEUROSCI.4478-06.2007 PMC6672798

[44] Kulich SM, Horbinski C, Patel M et al. 6-Hydroxydopamine induces mitochondrial ERK activation. Free Radic Biol Med. 2007;43:372–83 17602953 10.1016/j.freeradbiomed.2007.04.028 PMC2023873

[45] Tkacz JS, Lampen O. Tunicamycin inhibition of polyisoprenyl N-acetylglucosaminyl pyrophosphate formation in calf-liver microsomes. Biochem Biophys Res Commun. 1975;65:248–57 167767 10.1016/s0006-291x(75)80086-6

[46] Fliesler SJ, Rayborn ME, Hollyfield JG. Membrane morphogenesis in retinal rod outer segments: inhibition by tunicamycin. J Cell Biol. 1985;100:574–87 3155750 10.1083/jcb.100.2.574 PMC2113453

[47] Fliesler SJ, Rapp LM, Hollyfield JG. Photoreceptor-specific degeneration caused by tunicamycin. Nature. 1984;311:575–7 6332991 10.1038/311575a0

[48] Wang S, Liu Y, Tan JW et al. Tunicamycin-induced photoreceptor atrophy precedes degeneration of retinal capillaries with minimal effects on retinal ganglion and pigment epithelium cells. Exp Eye Res. 2019;187:107756 31421136 10.1016/j.exer.2019.107756 PMC7412575

[49] Tsubura A, Lai YC, Miki H et al. Review: animal models of N-methyl-N-nitrosourea-induced mammary cancer and retinal degeneration with special emphasis on therapeutic trials. In Vivo. 2011;25:11–22 21282729

[50] Maines MD, Sinclair P. Cobalt regulation of heme synthesis and degradation in avian embryo liver cell culture. J Biol Chem. 1977;252:219–23 556723

[51] Semenza GL. HIF-1 and mechanisms of hypoxia sensing. Curr Opin Cell Biol. 2001;13:167–71 11248550 10.1016/s0955-0674(00)00194-0

[52] Medrano MP, Pisera Fuster A, Sanchis PA et al. Characterization of proliferative, glial and angiogenic responses after a CoCl2 -induced injury of photoreceptor cells in the adult zebrafish retina. Eur J Neurosci. 2018;48:3019–42 30102439 10.1111/ejn.14113

[53] Medrano MP, Pisera-Fuster A, Bernabeu RO et al. P2X7 and A2Areceptor endogenous activation protects against neuronal death caused by CoCl2‐induced photoreceptor toxicity in the zebrafish retina. J Comp Neurol. 2020;528:2000–20 31997350 10.1002/cne.24869

[54] del Olmo-Aguado S, Núñez-Álvarez C, Ji D et al. RTP801 immunoreactivity in retinal ganglion cells and its down-regulation in cultured cells protect them from light and cobalt chloride. Brain Res Bull. 2013;98:132–44 23978538 10.1016/j.brainresbull.2013.08.002

[55] Youssef PN, Sheibani N, Albert DM. Retinal light toxicity. Eye (Lond). 2011;25:1–14 21178995 10.1038/eye.2010.149 PMC3144654

[56] Vihtelic TS, Hyde DR. Light-induced rod and cone cell death and regeneration in the adult albino zebrafish (Danio rerio) retina. J Neurobiol. 2000;44:289–307 10942883 10.1002/1097-4695(20000905)44:3<289::aid-neu1>3.0.co;2-h

[57] Thomas JL, Nelson CM, Luo X et al. Characterization of multiple light damage paradigms reveals regional differences in photoreceptor loss. Exp Eye Res. 2012;97:105–16 22425727 10.1016/j.exer.2012.02.004 PMC3329775

[58] Jopling C, Boue S, Izpisua Belmonte JC. Dedifferentiation, transdifferentiation and reprogramming: three routes to regeneration. Nat Rev Mol Cell Biol. 2011;12:79–89 21252997 10.1038/nrm3043

[59] Reh TA, Constantine-Paton M. Qualitative and quantitative measures of plasticity during the normal development of the Rana pipiens retinotectal projection. Brain Res. 1983;312:187–200 6606482 10.1016/0165-3806(83)90136-0

[60] Wan Y, Almeida AD, Rulands S et al. The ciliary marginal zone of the zebrafish retina: clonal and time-lapse analysis of a continuously growing tissue. Development. 2016;143:1099–107 26893352 10.1242/dev.133314 PMC4852496

[61] Todd L, Reh TA. Comparative biology of vertebrate retinal regeneration: restoration of vision through cellular reprogramming. Cold Spring Harb Perspect Biol. 2022;14:a040816 34580118 10.1101/cshperspect.a040816 PMC9248829

[62] Hitchcock P, Ochocinska M, Sieh A et al. Persistent and injury-induced neurogenesis in the vertebrate retina. Prog Retin Eye Res. 2004;23:183–94 15094130 10.1016/j.preteyeres.2004.01.001

[63] Amato MA, Arnault E, Perron M. Retinal stem cells in vertebrates: parallels and divergences. Int J Dev Biol. 2004;48:993–1001 15558490 10.1387/ijdb.041879ma

[64] Tropepe V, Coles BLK, Chiasson BJ et al. Retinal stem cells in the adult mammalian eye. Science. 2000;287:2032–6 10720333 10.1126/science.287.5460.2032

[65] Marcucci F, Murcia-Belmonte V, Wang Q et al. The ciliary margin zone of the mammalian retina generates retinal ganglion cells. Cell Rep. 2016;17:3153–64 28009286 10.1016/j.celrep.2016.11.016 PMC5234854

[66] Miyake A, Araki M. Retinal stem/progenitor cells in the ciliary marginal zone complete retinal regeneration: a study of retinal regeneration in a novel animal model. Dev Neurobiol. 2014;74:739–56 24488715 10.1002/dneu.22169

[67] Araki M. Regeneration of the amphibian retina: role of tissue interaction and related signaling molecules on RPE transdifferentiation. Develop Growth Differ. 2007;49:109–20 10.1111/j.1440-169X.2007.00911.x 17335432

[68] Del Rio-Tsonis K, Tsonis PA. Eye regeneration at the molecular age. Dev Dyn. 2003;226:211–24 12557200 10.1002/dvdy.10224

[69] Islam MR, Nakamura K, Casco-Robles MM et al. The newt reprograms mature RPE cells into a unique multipotent state for retinal regeneration. Sci Rep. 2014;4:6043 25116407 10.1038/srep06043 PMC4131214

[70] Yoshii C, Ueda Y, Okamoto M et al. Neural retinal regeneration in the anuran amphibian *Xenopus laevis* post-metamorphosis: transdifferentiation of retinal pigmented epithelium regenerates the neural retina. Dev Biol. 2007;303:45–56 17184765 10.1016/j.ydbio.2006.11.024

[71] Knight JK, Raymond PA. Retinal pigmented epithelium does not transdifferentiate in adult goldfish. J Neurobiol. 1995;27:447–56 7561826 10.1002/neu.480270402

[72] George SM, Lu F, Rao M et al. The retinal pigment epithelium: development, injury responses, and regenerative potential in mammalian and non-mammalian systems. Prog Retin Eye Res. 2021;85:100969 33901682 10.1016/j.preteyeres.2021.100969 PMC8536801

[73] Spence JR, Madhavan M, Aycinena JC et al. Retina regeneration in the chick embryo is not induced by spontaneous Mitf downregulation but requires FGF/FGFR/MEK/Erk dependent upregulation of Pax6. Mol Vis. 2007;13:57–65 17277739 PMC2503104

[74] Luz-Madrigal A, Grajales-Esquivel E, McCorkle A et al. Reprogramming of the chick retinal pigmented epithelium after retinal injury. BMC Biol. 2014;12:28 24742279 10.1186/1741-7007-12-28 PMC4026860

[75] Coulombre JL, Coulombre AJ. Regeneration of neural retina from the pigmented epithelium in the chick embryo. Dev Biol. 1965;12:79–92 5833111 10.1016/0012-1606(65)90022-9

[76] Coulombre JL, Coulombre AJ. Influence of mouse neural retina on regeneration of chick neural retina from chick embryonic pigmented epithelium. Nature. 1970;228:559–60 5472476 10.1038/228559a0

[77] Zhao S, Thornquist SC, Barnstable CJ. *In vitro* transdifferentiation of embryonic rat retinal pigment epithelium to neural retina. Brain Res. 1995;677:300–10 7552256 10.1016/0006-8993(95)00163-k

[78] Bernardos RL, Barthel LK, Meyers JR et al. Late-stage neuronal progenitors in the retina are radial Muller glia that function as retinal stem cells. J Neurosci. 2007;27:7028–40 17596452 10.1523/JNEUROSCI.1624-07.2007 PMC6672216

[79] Fausett BV, Goldman D. A role for 1 tubulin-expressing Muller glia in regeneration of the injured zebrafish retina. J Neurosci. 2006;26:6303–13 16763038 10.1523/JNEUROSCI.0332-06.2006 PMC6675181

[80] Fischer AJ, Reh TA. Müller glia are a potential source of neural regeneration in the postnatal chicken retina. Nat Neurosci. 2001;4:247–52 11224540 10.1038/85090

[81] Goldman D. Müller glial cell reprogramming and retina regeneration. Nat Rev Neurosci. 2014;15:431–42 24894585 10.1038/nrn3723 PMC4249724

[82] Hoang T, Wang J, Boyd P et al. Gene regulatory networks controlling vertebrate retinal regeneration. Science. 2020;370:eabb8598 33004674 10.1126/science.abb8598 PMC7899183

[83] Powell C, Grant AR, Cornblath E et al. Analysis of DNA methylation reveals a partial reprogramming of the Müller glia genome during retina regeneration. Proc Natl Acad Sci USA. 2013;110:19814–9 24248357 10.1073/pnas.1312009110 PMC3856824

[84] Nagashima M, Barthel LK, Raymond PA. A self-renewing division of zebrafish Müller glial cells generates neuronal progenitors that require N-cadherin to regenerate retinal neurons. Development. 2013;140:4510–21 24154521 10.1242/dev.090738 PMC3817940

[85] Lahne M et al. The regenerating adult zebrafish retina recapitulates developmental fate specification programs. Front Cell Dev Biol. 2020;8:617923 33598455 10.3389/fcell.2020.617923 PMC7882614

[86] Lenkowski JR, Raymond PA. Müller glia: stem cells for generation and regeneration of retinal neurons in teleost fish. Prog Retin Eye Res. 2014;40:94–123 24412518 10.1016/j.preteyeres.2013.12.007 PMC3999222

[87] Garcia-Garcia D, Locker M, Perron M. Update on Muller glia regenerative potential for retinal repair. Curr Opin Genet Dev. 2020;64:52–9 32619816 10.1016/j.gde.2020.05.025

[88] Todd L, Squires N, Suarez L et al. Jak/Stat signaling regulates the proliferation and neurogenic potential of Muller glia-derived progenitor cells in the avian retina. Sci Rep. 2016;6:35703 27759082 10.1038/srep35703 PMC5069623

[89] Todd L, Volkov LI, Zelinka C et al. Heparin-binding EGF-like growth factor (HB-EGF) stimulates the proliferation of Müller glia-derived progenitor cells in avian and murine retinas. Mol Cell Neurosci. 2015;69:54–64 26500021 10.1016/j.mcn.2015.10.004 PMC4658256

[90] Jorstad NL, Wilken MS, Grimes WN et al. Stimulation of functional neuronal regeneration from Müller glia in adult mice. Nature. 2017;548:103–7 28746305 10.1038/nature23283 PMC5991837

[91] Joly S, Pernet V, Samardzija M et al. Pax6-positive müller glia cells express cell cycle markers but do not proliferate after photoreceptor injury in the mouse retina. Glia. 2011;59:1033–46 21500284 10.1002/glia.21174

[92] Reyes-Aguirre LI, Lamas M. Oct4 methylation-mediated silencing as an epigenetic barrier preventing Muller glia dedifferentiation in a murine model of retinal injury. Front Neurosci. 2016;10:523 27895551 10.3389/fnins.2016.00523 PMC5108807

[93] Morris AC, Scholz T, Fadool JM. Rod progenitor cells in the mature zebrafish retina. Adv Exp Med Biol. 2008;613:361–8 18188965 10.1007/978-0-387-74904-4_42 PMC2846520

[94] Otteson DC, Hitchcock PF. Stem cells in the teleost retina: persistent neurogenesis and injury-induced regeneration. Vis Res. 2003;43:927–36 12668062 10.1016/s0042-6989(02)00400-5

[95] Stenkamp DL. The rod photoreceptor lineage of teleost fish. Prog Retin Eye Res. 2011;30:395–404 21742053 10.1016/j.preteyeres.2011.06.004 PMC3196835

[96] Montgomery JE, Parsons MJ, Hyde DR. A novel model of retinal ablation demonstrates that the extent of rod cell death regulates the origin of the regenerated zebrafish rod photoreceptors. J Comp Neurol. 2010;518:800–14 20058308 10.1002/cne.22243 PMC3656417

[97] Turkalj B, Quallich D, Bessert DA et al. Development and characterization of a chronic photoreceptor degeneration model in adult zebrafish that does not trigger a regenerative response. Exp Eye Res. 2021;209:108630 34029596 10.1016/j.exer.2021.108630 PMC8595574

[98] Schmitner N, Recheis C, Thönig J et al. Differential responses of neural retina progenitor populations to chronic hyperglycemia. Cell. 2021;10:3265 10.3390/cells10113265 PMC8622914 34831487

[99] Ochocinska MJ, Hitchcock PF. Dynamic expression of the basic helix-loop-helix transcription factor neuroD in the rod and cone photoreceptor lineages in the retina of the embryonic and larval zebrafish. J Comp Neurol. 2007;501:1–12 17206615 10.1002/cne.21150

[100] Morris AC, Scholz TL, Brockerhoff SE et al. Genetic dissection reveals two separate pathways for rod and cone regeneration in the teleost retina. Dev Neurobiol. 2008;68:605–19 18265406 10.1002/dneu.20610 PMC2801137

[101] Goldman JA, Poss KD. Gene regulatory programmes of tissue regeneration. Nat Rev Genet. 2020;21:511–25 32504079 10.1038/s41576-020-0239-7 PMC7448550

[102] Cheon EW, Kaneko Y, Saito T. Regeneration of the newt retina: order of appearance of photoreceptors and ganglion cells. J Comp Neurol. 1998;396:267–74 9634147

[103] Hayes S, Nelson BR, Buckingham B et al. Notch signaling regulates regeneration in the avian retina. Dev Biol. 2007;312:300–11 18028900 10.1016/j.ydbio.2007.09.046 PMC2170876

[104] Todd L, Suarez L, Quinn C et al. Retinoic acid-signaling regulates the proliferative and neurogenic capacity of Müller glia-derived progenitor cells in the avian retina. Stem Cells. 2018;36:392–405 29193451 10.1002/stem.2742 PMC5823757

[105] Nishida A, Furukawa A, Koike C et al. Otx2 homeobox gene controls retinal photoreceptor cell fate and pineal gland development. Nat Neurosci. 2003;6:1255–63 14625556 10.1038/nn1155

[106] Kay JN, Finger-Baier KC, Roeser T et al. Retinal ganglion cell genesis requires lakritz, a zebrafish atonal homolog. Neuron. 2001;30:725–36 11430806 10.1016/s0896-6273(01)00312-9

[107] Ramachandran R, Fausett BV, Goldman D. Ascl1a regulates Müller glia dedifferentiation and retinal regeneration through a Lin-28-dependent, let-7 microRNA signalling pathway. Nat Cell Biol. 2010;12:1101–7 20935637 10.1038/ncb2115 PMC2972404

[108] Ambros V. MicroRNAs and developmental timing. Curr Opin Genet Dev. 2011;21:511–7 21530229 10.1016/j.gde.2011.04.003 PMC3149784

[109] Decembrini S, Bressan D, Vignali R et al. MicroRNAs couple cell fate and developmental timing in retina. Proc Natl Acad Sci USA. 2009;106:21179–84 19965369 10.1073/pnas.0909167106 PMC2781736

[110] La Torre A, Georgi S, Reh TA. Conserved microRNA pathway regulates developmental timing of retinal neurogenesis. Proc Natl Acad Sci USA. 2013;110:E2362–70 23754433 10.1073/pnas.1301837110 PMC3696811

[111] Unternaehrer JJ, Zhao R, Kim K et al. The epithelial-mesenchymal transition factor SNAIL paradoxically enhances reprogramming. Stem Cell Rep. 2014;3:691–8 10.1016/j.stemcr.2014.09.008 PMC4235745 25316190

[112] Sharma P, Gupta S, Chaudhary M et al. Biphasic role of Tgf-β signaling during Müller glia reprogramming and retinal regeneration in zebrafish. iScience. 2020;23:100817 32004993 10.1016/j.isci.2019.100817 PMC6994856

[113] Brabletz S, Brabletz T. The ZEB/miR-200 feedback loop--a motor of cellular plasticity in development and cancer? EMBO Rep. 2010;11:670–7 20706219 10.1038/embor.2010.117 PMC2933868

[114] Mani SA, Guo W, Liao MJ et al. The epithelial-mesenchymal transition generates cells with properties of stem cells. Cell. 2008;133:704–15 18485877 10.1016/j.cell.2008.03.027 PMC2728032

[115] Shuang ZY, Wu WC, Xu J et al. Transforming growth factor-β1-induced epithelial–mesenchymal transition generates ALDH-positive cells with stem cell properties in cholangiocarcinoma. Cancer Lett. 2014;354:320–8 25194504 10.1016/j.canlet.2014.08.030

[116] Iribarne M. Inflammation induces zebrafish regeneration. Neural Regen Res. 2021;16:1693–701 33510057 10.4103/1673-5374.306059 PMC8328752

[117] Nagashima M, Hitchcock PF. Inflammation regulates the multi-step process of retinal regeneration in zebrafish. Cell. 2021;10:783 10.3390/cells10040783 PMC8066466 33916186

[118] Serhan CN, Brain SD, Buckley CD et al. Resolution of inflammation: state of the art, definitions and terms. FASEB J. 2007;21:325–32 17267386 10.1096/fj.06-7227rev PMC3119634

[119] Lee J, Sayed N, Hunter A et al. Activation of innate immunity is required for efficient nuclear reprogramming. Cell. 2012;151:547–58 23101625 10.1016/j.cell.2012.09.034 PMC3506423

[120] Michael S et al. Inflammation shapes stem cells and stemness during infection and beyond. Front Cell Dev Biol. 2016;4:118 27853732 10.3389/fcell.2016.00118 PMC5089974

[121] Clark BS, Stein-O’Brien GL, Shiau F et al. Single-cell RNA-seq analysis of retinal development identifies NFI factors as regulating mitotic exit and late-born cell specification. Neuron. 2019;102:1111–1126.e5 31128945 10.1016/j.neuron.2019.04.010 PMC6768831

[122] Palazzo I et al. NF-kappaB signaling regulates the formation of proliferating Muller glia-derived progenitor cells in the avian retina. Development. 2020;147:dev183418 32291273 10.1242/dev.183418 PMC7325431

[123] Haynes T, Luz-Madrigal A, Reis ES et al. Complement anaphylatoxin C3a is a potent inducer of embryonic chick retina regeneration. Nat Commun. 2013;4:2312 23942241 10.1038/ncomms3312 PMC3753547

[124] Conedera FM, Pousa AMQ, Mercader N et al. Retinal microglia signaling affects Müller cell behavior in the zebrafish following laser injury induction. Glia. 2019;67:1150–66 30794326 10.1002/glia.23601

[125] White DT, Sengupta S, Saxena MT et al. Immunomodulation-accelerated neuronal regeneration following selective rod photoreceptor cell ablation in the zebrafish retina. Proc Natl Acad Sci USA. 2017;114:E3719–28 28416692 10.1073/pnas.1617721114 PMC5422825

[126] Zhang Z, Hou H, Yu S et al. Inflammation-induced mammalian target of rapamycin signaling is essential for retina regeneration. Glia. 2020;68:111–27 31444939 10.1002/glia.23707

[127] Lu F, Leach LL, Gross JM. mTOR activity is essential for retinal pigment epithelium regeneration in zebrafish. PLoS Genet. 2022;18:e1009628 35271573 10.1371/journal.pgen.1009628 PMC8939802

[128] Leach LL et al. The immune response is a critical regulator of zebrafish retinal pigment epithelium regeneration. Proc Natl Acad Sci USA. 2021;118:e2017198118 34006636 10.1073/pnas.2017198118 PMC8166181

[129] Todd L, Finkbeiner C, Wong CK et al. Microglia suppress Ascl1-induced retinal regeneration in mice. Cell Rep. 2020;33:108507 33326790 10.1016/j.celrep.2020.108507

[130] Nagashima M, D'Cruz TS, Danku AE et al. Midkine-a is required for cell cycle progression of Müller glia during neuronal regeneration in the vertebrate retina. J Neurosci. 2020;40:1232–47 31882403 10.1523/JNEUROSCI.1675-19.2019 PMC7002140

[131] Iribarne M, Hyde DR, Masai I. TNFalpha induces Muller glia to transition from non-proliferative gliosis to a regenerative response in mutant zebrafish presenting chronic photoreceptor degeneration. Front Cell Dev Biol. 2019;7:296 31998714 10.3389/fcell.2019.00296 PMC6962764

[132] Silva NJ, Nagashima M, Li J et al. Inflammation and matrix metalloproteinase 9 (Mmp-9) regulate photoreceptor regeneration in adult zebrafish. Glia. 2020;68:1445–65 32034934 10.1002/glia.23792 PMC7317489

[133] Kaur S, Gupta S, Chaudhary M et al. let-7 MicroRNA-mediated regulation of Shh signaling and the gene regulatory network is essential for retina regeneration. Cell Rep. 2018;23:1409–23 29719254 10.1016/j.celrep.2018.04.002 PMC5946716

[134] Naitoh H, Suganuma Y, Ueda Y et al. Upregulation of matrix metalloproteinase triggers transdifferentiation of retinal pigmented epithelial cells in *Xenopus laevis*: a link between inflammatory response and regeneration. Dev Neurobiol. 2017;77:1086–100 28371543 10.1002/dneu.22497

[135] Nelson CM, Ackerman KM, O'Hayer P et al. Tumor necrosis factor-alpha is produced by dying retinal neurons and is required for Muller glia proliferation during zebrafish retinal regeneration. J Neurosci. 2013;33:6524–39 23575850 10.1523/JNEUROSCI.3838-12.2013 PMC3740543

[136] Wan J, Zhao XF, Vojtek A et al. Retinal injury, growth factors, and cytokines converge on beta-catenin and pStat3 signaling to stimulate retina regeneration. Cell Rep. 2014;9:285–97 25263555 10.1016/j.celrep.2014.08.048 PMC4194164

[137] Zhao XF, Wan J, Powell C et al. Leptin and IL-6 family cytokines synergize to stimulate Müller glia reprogramming and retina regeneration. Cell Rep. 2014;9:272–84 25263554 10.1016/j.celrep.2014.08.047 PMC4194149

[138] Conedera FM, Quintela Pousa AM, Presby DM et al. Diverse signaling by TGFβ isoforms in response to focal injury is associated with either retinal regeneration or reactive gliosis. Cell Mol Neurobiol. 2021;41:43–62 32219603 10.1007/s10571-020-00830-5 PMC7811507

[139] Lee MS, Wan J, Goldman D. Tgfb3 collaborates with PP2A and Notch signaling pathways to inhibit retina regeneration. elife. 2020;9:e55137 32396062 10.7554/eLife.55137 PMC7250569

[140] Braunger BM, Pielmeier S, Demmer C et al. TGF-signaling protects retinal neurons from programmed cell death during the development of the mammalian eye. J Neurosci. 2013;33:14246–58 23986258 10.1523/JNEUROSCI.0991-13.2013 PMC6618509

[141] Ma W, Silverman SM, Zhao L et al. Absence of TGFβ signaling in retinal microglia induces retinal degeneration and exacerbates choroidal neovascularization. elife. 2019;8:e42049 30666961 10.7554/eLife.42049 PMC6342522

[142] Conedera FM, Pousa AMQ, Mercader N et al. The TGFβ/Notch axis facilitates Müller cell-to-epithelial transition to ultimately form a chronic glial scar. Mol Neurodegener. 2021;16:69 34593012 10.1186/s13024-021-00482-z PMC8482586

[143] Sharma P, Gupta S, Chaudhary M et al. Oct4 mediates Müller glia reprogramming and cell cycle exit during retina regeneration in zebrafish. Life Sci Alliance. 2019;2:e201900548 31594822 10.26508/lsa.201900548 PMC6784428

[144] Mitra S, Sharma P, Kaur S et al. Dual regulation of lin28a by Myc is necessary during zebrafish retina regeneration. J Cell Biol. 2019;218:489–507 30606747 10.1083/jcb.201802113 PMC6363449

[145] Gorsuch RA, Lahne M, Yarka CE et al. Sox2 regulates Müller glia reprogramming and proliferation in the regenerating zebrafish retina via Lin28 and Ascl1a. Exp Eye Res. 2017;161:174–92 28577895 10.1016/j.exer.2017.05.012 PMC5554723

[146] Reinhardt R, Centanin L, Tavhelidse T et al. Sox2, Tlx, Gli3, and Her9 converge on Rx2 to define retinal stem cells *in vivo*. EMBO J. 2015;34:1572–88 25908840 10.15252/embj.201490706 PMC4474531

[147] Lust K, Wittbrodt J. Activating the regenerative potential of Muller glia cells in a regeneration-deficient retina. elife. 2018;7:32319 10.7554/eLife.32319 PMC5815849 29376827

[148] Ma W, Yan RT, Li X et al. Reprogramming retinal pigment epithelium to differentiate toward retinal neurons with Sox2. Stem Cells. 2009;27:1376–87 19489100 10.1002/stem.48 PMC2746631

[149] Elsaeidi F, Macpherson P, Mills EA et al. Notch suppression collaborates with Ascl1 and Lin28 to unleash a regenerative response in fish retina, but not in mice. J Neurosci. 2018;38:2246–61 29378863 10.1523/JNEUROSCI.2126-17.2018 PMC5830513

[150] Krohne TU, Westenskow PD, Kurihara T et al. Generation of retinal pigment epithelial cells from small molecules and OCT4 reprogrammed human induced pluripotent stem cells. Stem Cells Transl Med. 2012;1:96–109 22532929 10.5966/sctm.2011-0057 PMC3328503

[151] Zelinka CP, Volkov L, Goodman ZA et al. mTor signaling is required for the formation of proliferating Müller glia-derived progenitor cells in the chick retina. Development. 2016;143:1859–73 27068108 10.1242/dev.133215 PMC4920162

[152] Gupta S et al. Temporal regulation of Pten is essential for retina regeneration in zebrafish. bioRxiv. 2021; 2021.07.09.451767

[153] Masson C, García-García D, Bitard J et al. Yap haploinsufficiency leads to Muller cell dysfunction and late-onset cone dystrophy. Cell Death Dis. 2020;11:631 32801350 10.1038/s41419-020-02860-9 PMC7429854

[154] Hamon A, García-García D, Ail D et al. Linking YAP to Müller glia quiescence exit in the degenerative retina. Cell Rep. 2019;27:1712–1725.e6 31067458 10.1016/j.celrep.2019.04.045

[155] Rueda EM, Hall BM, Hill MC et al. The hippo pathway blocks mammalian retinal Müller glial cell reprogramming. Cell Rep. 2019;27:1637–1649.e6 31067451 10.1016/j.celrep.2019.04.047 PMC6521882

[156] Lourenco R et al. Yap regulates Muller glia reprogramming in damaged zebrafish retinas. Front Cell Dev Biol. 2021;9:667796 34616723 10.3389/fcell.2021.667796 PMC8488126

[157] Asaoka Y, Hata S, Namae M et al. The hippo pathway controls a switch between retinal progenitor cell proliferation and photoreceptor cell differentiation in zebrafish. PLoS One. 2014;9:e97365 24828882 10.1371/journal.pone.0097365 PMC4020862

[158] Locker M, Agathocleous M, Amato MA et al. Hedgehog signaling and the retina: insights into the mechanisms controlling the proliferative properties of neural precursors. Genes Dev. 2006;20:3036–48 17079690 10.1101/gad.391106 PMC1620016

[159] Thomas JL, Morgan GW, Dolinski KM, Thummel R. Characterization of the pleiotropic roles of sonic hedgehog during retinal regeneration in adult zebrafish. Exp Eye Res. 2018;166:106–15 29030175 10.1016/j.exer.2017.10.003 PMC5756498

[160] Spence JR, Madhavan M, Ewing JD et al. The hedgehog pathway is a modulator of retina regeneration. Development. 2004;131:4607–21 15342484 10.1242/dev.01298

[161] Gu D, Wang S, Zhang S et al. Directed transdifferentiation of Müller glial cells to photoreceptors using the sonic hedgehog signaling pathway agonist purmorphamine. Mol Med Rep. 2017;16:7993–8002 28983586 10.3892/mmr.2017.7652 PMC5779882

[162] Wu J, Zhang S, Sun X. Neuroprotective effect of upregulated sonic hedgehog in retinal ganglion cells following chronic ocular hypertension. Invest Ophthalmol Vis Sci. 2010;51:2986–92 20071678 10.1167/iovs.09-4151

[163] Majidinia M, Aghazadeh J, Jahanban-Esfahlani R et al. The roles of Wnt/β‐catenin pathway in tissue development and regenerative medicine. J Cell Physiol. 2018;233:5598–612 29150936 10.1002/jcp.26265

[164] Ramachandran R, Zhao XF, Goldman D. Ascl1a/Dkk/beta-catenin signaling pathway is necessary and glycogen synthase kinase-3beta inhibition is sufficient for zebrafish retina regeneration. Proc Natl Acad Sci USA. 2011;108:15858–63 21911394 10.1073/pnas.1107220108 PMC3179085

[165] Ramachandran R, Zhao XF, Goldman D. Insm1a-mediated gene repression is essential for the formation and differentiation of Müller glia-derived progenitors in the injured retina. Nat Cell Biol. 2012;14:1013–23 23000964 10.1038/ncb2586 PMC3463712

[166] Steinfeld J, Steinfeld I, Bausch A et al. BMP-induced reprogramming of the neural retina into retinal pigment epithelium requires Wnt signalling. Biol Open. 2017;6:979–92 28546339 10.1242/bio.018739 PMC5550904

[167] Osakada F, Ooto S, Akagi T et al. Wnt signaling promotes regeneration in the retina of adult mammals. J Neurosci. 2007;27:4210–9 17428999 10.1523/JNEUROSCI.4193-06.2007 PMC6672527

[168] Sanges D, Romo N, Simonte G et al. Wnt/β-catenin signaling triggers neuron reprogramming and regeneration in the mouse retina. Cell Rep. 2013;4:271–86 23850287 10.1016/j.celrep.2013.06.015

[169] Yao K, Qiu S, Tian L et al. Wnt regulates proliferation and neurogenic potential of Müller glial cells via a Lin28/let-7 miRNA-dependent pathway in adult mammalian retinas. Cell Rep. 2016;17:165–78 27681429 10.1016/j.celrep.2016.08.078 PMC5076887

[170] Yao K, Qiu S, Wang YV et al. Restoration of vision after de novo genesis of rod photoreceptors in mammalian retinas. Nature. 2018;560:484–8 30111842 10.1038/s41586-018-0425-3 PMC6107416

[171] Wen X, Jiao L, Tan H. MAPK/ERK pathway as a central regulator in vertebrate organ regeneration. Int J Mol Sci. 2022;23:1464 35163418 10.3390/ijms23031464 PMC8835994

[172] Fischer AJ, Scott MA, Tuten W. Mitogen-activated protein kinase-signaling stimulates Müller glia to proliferate in acutely damaged chicken retina. Glia. 2009;57:166–81 18709648 10.1002/glia.20743 PMC2774719

[173] Agca C, Gubler A, Traber G et al. p38 MAPK signaling acts upstream of LIF-dependent neuroprotection during photoreceptor degeneration. Cell Death Dis. 2013;4:e785 24008729 10.1038/cddis.2013.323 PMC3789181

[174] Gallina D, Zelinka C, Fischer AJ. Glucocorticoid receptors in the retina, Müller glia and the formation of Müller glia-derived progenitors. Development. 2014;141:3340–51 25085975 10.1242/dev.109835 PMC4199119

[175] Wenzel A, Grimm C, Seeliger MW et al. Prevention of photoreceptor apoptosis by activation of the glucocorticoid receptor. Invest Ophthalmol Vis Sci. 2001;42:1653–9 11381074

[176] Grise KN et al. Glucocorticoid agonists enhance retinal stem cell self-renewal and proliferation. Stem Cell Res Ther. 2021;12:83 33494791 10.1186/s13287-021-02136-9 PMC7831262

[177] Sahu A, Devi S, Jui J et al. Notch signaling via Hey1 and Id2b regulates Müller glia's regenerative response to retinal injury. Glia. 2021;69:2882–98 34415582 10.1002/glia.24075 PMC8502208

[178] Conner C, Ackerman KM, Lahne M et al. Repressing Notch signaling and expressing TNF are sufficient to mimic retinal regeneration by inducing Muller glial proliferation to generate committed progenitor cells. J Neurosci. 2014;34:14403–19 25339752 10.1523/JNEUROSCI.0498-14.2014 PMC4205560

[179] Wan J, Goldman D. Opposing actions of Fgf8a on Notch signaling distinguish two Muller glial cell populations that contribute to retina growth and regeneration. Cell Rep. 2017;19:849–62 28445734 10.1016/j.celrep.2017.04.009 PMC5467687

[180] Nakamura K, Chiba C. Evidence for Notch signaling involvement in retinal regeneration of adult newt. Brain Res. 2007;1136:28–42 17217933 10.1016/j.brainres.2006.12.032

[181] de Melo J, Zibetti C, Clark BS et al. Lhx2 is an essential factor for retinal gliogenesis and Notch signaling. J Neurosci. 2016;36:2391–405 26911688 10.1523/JNEUROSCI.3145-15.2016 PMC4764661

[182] Xie J, Huo S, Li Y et al. Olfactory ensheathing cells inhibit gliosis in retinal degeneration by downregulation of the Müller cell Notch signaling pathway. Cell Transplant. 2017;26:967–82 28185609 10.3727/096368917X694994 PMC5657754

[183] Niu L, Fang Y, Yao X et al. TNFα activates MAPK and Jak-Stat pathways to promote mouse Müller cell proliferation. Exp Eye Res. 2021;202:108353 33171193 10.1016/j.exer.2020.108353

[184] Jorstad NL, Wilken MS, Todd L et al. STAT signaling modifies Ascl1 chromatin binding and limits neural regeneration from Muller glia in adult mouse retina. Cell Rep. 2020;30:2195–2208.e5 32075759 10.1016/j.celrep.2020.01.075 PMC7148114

[185] Basinski BW, Balikov DA, Aksu M et al. Ubiquitous chromatin modifiers in congenital retinal diseases: implications for disease modeling and regenerative medicine. Trends Mol Med. 2021;27:365–78 33573910 10.1016/j.molmed.2021.01.001 PMC8034778

[186] Luz-Madrigal A, Grajales-Esquivel E, Tangeman J et al. DNA demethylation is a driver for chick retina regeneration. Epigenetics. 2020;15:998–1019 32290791 10.1080/15592294.2020.1747742 PMC7518676

[187] Dvoriantchikova G, Seemungal RJ, Ivanov D. Development and epigenetic plasticity of murine Müller glia. Biochim Biophys Acta Mol Cell Res. 2019;1866:1584–94 31276697 10.1016/j.bbamcr.2019.06.019 PMC6684404

[188] Lin S, Guo J, Chen S. Transcriptome and DNA Methylome signatures associated with retinal Müller glia development, injury response, and aging. Invest Ophthalmol Vis Sci. 2019;60:4436–50 31652328 10.1167/iovs.19-27361

[189] Mitra S, Sharma P, Kaur S et al. Histone deacetylase-mediated Müller glia reprogramming through Her4.1-Lin28a axis is essential for retina regeneration in zebrafish. iScience. 2018;7:68–84 30267687 10.1016/j.isci.2018.08.008 PMC6135741

[190] Kara N, Kent MR, Didiano D et al. The miR-216a-Dot1l regulatory axis is necessary and sufficient for Müller glia reprogramming during retina regeneration. Cell Rep. 2019;28:2037–2047.e4 31433981 10.1016/j.celrep.2019.07.061 PMC6750267

[191] Dvoriantchikova G, Seemungal RJ, Ivanov D. The epigenetic basis for the impaired ability of adult murine retinal pigment epithelium cells to regenerate retinal tissue. Sci Rep. 2019;9:3860 30846751 10.1038/s41598-019-40262-w PMC6405859

[192] Pollak J, Wilken MS, Ueki Y et al. ASCL1 reprograms mouse Muller glia into neurogenic retinal progenitors. Development. 2013;140:2619–31 23637330 10.1242/dev.091355 PMC3666387

[193] Ueki Y, Wilken MS, Cox KE et al. Transgenic expression of the proneural transcription factor Ascl1 in Müller glia stimulates retinal regeneration in young mice. Proc Natl Acad Sci USA. 2015;112:13717–22 26483457 10.1073/pnas.1510595112 PMC4640735

[194] Sun LF, Chen XJ, Jin ZB. Emerging roles of non-coding RNAs in retinal diseases: a review. Clin Exp Ophthalmol. 2020;48:1085–101 32519377 10.1111/ceo.13806

[195] Raeisossadati R, Ferrari MFR, Kihara AH et al. Epigenetic regulation of retinal development. Epigenetics Chromatin. 2021;14:11 33563331 10.1186/s13072-021-00384-w PMC7871400

[196] Ha M, Kim VN. Regulation of microRNA biogenesis. Nat Rev Mol Cell Biol. 2014;15:509–24 25027649 10.1038/nrm3838

[197] Quintero H, Lamas M. microRNA expression in the neural retina: focus on Müller glia. J Neurosci Res. 2018;96:362–70 29030949 10.1002/jnr.24181

[198] Giraldez AJ, Cinalli RM, Glasner ME et al. MicroRNAs regulate brain morphogenesis in zebrafish. Science. 2005;308:833–8 15774722 10.1126/science.1109020

[199] Decembrini S, Andreazzoli M, Barsacchi G et al. Dicer inactivation causes heterochronic retinogenesis in *Xenopus laevis*. Int J Dev Biol. 2008;52:1099–103 18956342 10.1387/ijdb.082646sd

[200] Damiani D, Alexander JJ, O’Rourke JR et al. Dicer inactivation leads to progressive functional and structural degeneration of the mouse retina. J Neurosci. 2008;28:4878–87 18463241 10.1523/JNEUROSCI.0828-08.2008 PMC3325486

[201] Rajaram K, Harding RL, Bailey T et al. Dynamic miRNA expression patterns during retinal regeneration in zebrafish: reduced dicer or miRNA expression suppresses proliferation of Müller glia‐derived neuronal progenitor cells. Dev Dyn. 2014;243:1591–605 25220904 10.1002/dvdy.24188 PMC4237695

[202] Rajaram K, Harding RL, Hyde DR et al. miR-203 regulates progenitor cell proliferation during adult zebrafish retina regeneration. Dev Biol. 2014;392:393–403 24858486 10.1016/j.ydbio.2014.05.005 PMC4104251

[203] Wohl SG, Hooper MJ, Reh TA. MicroRNAs miR-25, let-7 and miR-124 regulate the neurogenic potential of Muller glia in mice. Development. 2019;146:dev179556 31383796 10.1242/dev.179556 PMC6765125

[204] Monaghan JR, Stier AC, Michonneau F et al. Experimentally induced metamorphosis in axolotls reduces regenerative rate and fidelity. Regeneration (Oxf). 2014;1:2–14 27499857 10.1002/reg2.8 PMC4895291

[205] Godwin JW, Rosenthal N. Scar-free wound healing and regeneration in amphibians: immunological influences on regenerative success. Differentiation. 2014;87:66–75 24565918 10.1016/j.diff.2014.02.002

[206] Dzulova D et al. Incomplete recovery of zebrafish retina following cryoinjury. Cell. 2022;11:1373 10.3390/cells11081373 PMC9030934 35456052

[207] Brandli A, Dudczig S, Currie PD et al. Photoreceptor ablation following ATP induced injury triggers Müller glia driven regeneration in zebrafish. Exp Eye Res. 2021;207:108569 33839111 10.1016/j.exer.2021.108569

[208] Tappeiner C, Maurer E, Sallin P et al. Inhibition of the TGFβ pathway enhances retinal regeneration in adult zebrafish. PLoS One. 2016;11:e0167073 27880821 10.1371/journal.pone.0167073 PMC5120850

[209] Braisted JE, Raymond PA. Regeneration of dopaminergic neurons in goldfish retina. Development. 1992;114:913–9 1618153 10.1242/dev.114.4.913

[210] Hitchcock PF, Vanderyt JT. Regeneration of the dopamine-cell mosaic in the retina of the goldfish. Vis Neurosci. 1994;11:209–17 7911678 10.1017/s0952523800001577

[211] Yazulla S, Lin ZS, Studholme KM. Dopaminergic control of light-adaptive synaptic plasticity and role in goldfish visual behavior. Vis Res. 1996;36:4045–57 9068857 10.1016/s0042-6989(96)00128-9

[212] Braisted JE, Raymond PA. Continued search for the cellular signals that regulate regeneration of dopaminergic neurons in goldfish retina. Brain Res Dev Brain Res. 1993;76:221–32 8149588 10.1016/0165-3806(93)90210-2

[213] Cocchiaro P, di Donato V, Rubbini D et al. Intravitreal administration of rhNGF enhances regenerative processes in a zebrafish model of retinal degeneration. Front Pharmacol. 2022;13:822359 35330834 10.3389/fphar.2022.822359 PMC8940169

[214] Stulberg J, Tropepe V. *In situ* quantification and isolation of Müller glial cells by fluorescence-activated cell sorting from the regenerating larval zebrafish retina. Methods Mol Biol. 2022;2429:345–56 35507172 10.1007/978-1-0716-1979-7_22

[215] Yoshimatsu T, D’Orazi FD, Gamlin CR et al. Presynaptic partner selection during retinal circuit reassembly varies with timing of neuronal regeneration in vivo. Nat Commun. 2016;7:10590 26838932 10.1038/ncomms10590 PMC4742908

[216] Hagerman GF, Noel NCL, Cao SY et al. Rapid recovery of visual function associated with blue cone ablation in zebrafish. PLoS One. 2016;11:e0166932 27893779 10.1371/journal.pone.0166932 PMC5125653

[217] Pisharath H. Validation of nitroreductase, a prodrug-activating enzyme, mediated cell death in embryonic zebrafish (Danio rerio). Comp Med. 2007;57:241–6 17605338

[218] Zhao XF, Ellingsen S, Fjose A. Labelling and targeted ablation of specific bipolar cell types in the zebrafish retina. BMC Neurosci. 2009;10:107 19712466 10.1186/1471-2202-10-107 PMC3224687

[219] Nakao T, Tsujikawa M, Notomi S et al. The role of mislocalized phototransduction in photoreceptor cell death of retinitis pigmentosa. PLoS One. 2012;7:e32472 22485131 10.1371/journal.pone.0032472 PMC3317642

[220] Santhanam A, Shihabeddin E, Atkinson JA et al. A zebrafish model of retinitis pigmentosa shows continuous degeneration and regeneration of rod photoreceptors. Cell. 2020;9:2242 10.3390/cells9102242 PMC7599532 33036185

[221] Ali Z, Zang J, Lagali N et al. Photoreceptor degeneration accompanies vascular changes in a zebrafish model of diabetic retinopathy. Invest Ophthalmol Vis Sci. 2020;61:43 10.1167/iovs.61.2.43 PMC7329949 32106290

[222] Lodd E et al. The combination of loss of glyoxalase1 and obesity results in hyperglycemia. JCI Insight. 2019;4:e126154 31217350 10.1172/jci.insight.126154 PMC6629122

[223] Wiggenhauser LM, Qi H, Stoll SJ et al. Activation of retinal angiogenesis in Hyperglycemicpdx1−/−zebrafish mutants. Diabetes. 2020;69:1020–31 32139597 10.2337/db19-0873

[224] Biehlmaier O, Neuhauss SC, Kohler K. Double cone dystrophy and RPE degeneration in the retina of the zebrafish gnn mutant. Invest Ophthalmol Vis Sci. 2003;44:1287–98 12601061 10.1167/iovs.02-0363

[225] Hanovice NJ, Leach LL, Slater K et al. Regeneration of the zebrafish retinal pigment epithelium after widespread genetic ablation. PLoS Genet. 2019;15:e1007939 30695061 10.1371/journal.pgen.1007939 PMC6368336

[226] Viringipurampeer IA, Shan X, Gregory-Evans K et al. Rip3 knockdown rescues photoreceptor cell death in blind pde6c zebrafish. Cell Death Differ. 2014;21:665–75 24413151 10.1038/cdd.2013.191 PMC3978298

[227] Veth KN, Willer JR, Collery RF et al. Mutations in zebrafish lrp2 result in adult-onset ocular pathogenesis that models myopia and other risk factors for glaucoma. PLoS Genet. 2011;7:e1001310 21379331 10.1371/journal.pgen.1001310 PMC3040661

[228] Link BA, Gray MP, Smith RS et al. Intraocular pressure in zebrafish: comparison of inbred strains and identification of a reduced melanin mutant with raised IOP. Invest Ophthalmol Vis Sci. 2004;45:4415–22 15557450 10.1167/iovs.04-0557

[229] Martinez-De Luna RI, Zuber ME. Rod-specific ablation using the nitroreductase/metronidazole system to investigate regeneration in *Xenopus*. Cold Spring Harb Protoc. 2018;2018:100974 10.1101/pdb.prot100974 29789402

[230] Chesneau A, Bronchain O, Perron M. Conditional chemogenetic ablation of photoreceptor cells in *Xenopus* retina. Methods Mol Biol. 2018;1865:133–46 30151764 10.1007/978-1-4939-8784-9_10

[231] Ropelewski P, Imanishi Y. Disrupted plasma membrane protein homeostasis in a *Xenopus laevis* model of retinitis pigmentosa. J Neurosci. 2019;39:5581–93 31061086 10.1523/JNEUROSCI.3025-18.2019 PMC6616295

[232] Feehan JM, Chiu CN, Stanar P et al. Modeling dominant and recessive forms of retinitis pigmentosa by editing three rhodopsin-encoding genes in *Xenopus laevis* using Crispr/Cas9. Sci Rep. 2017;7:6920 28761125 10.1038/s41598-017-07153-4 PMC5537283

[233] Parain K, Lourdel S, Donval A et al. CRISPR/Cas9-mediated models of retinitis pigmentosa reveal differential proliferative response of Muller cells between *Xenopus laevis* and *Xenopus tropicalis*. Cell. 2022;11:807 10.3390/cells11050807 PMC8909648 35269429

[234] Todd L, Palazzo I, Squires N et al. BMP- and TGFβ-signaling regulate the formation of Müller glia-derived progenitor cells in the avian retina. Glia. 2017;65:1640–55 28703293 10.1002/glia.23185 PMC5628513

[235] Nagar S, Krishnamoorthy V, Cherukuri P et al. Early remodeling in an inducible animal model of retinal degeneration. Neuroscience. 2009;160:517–29 19272416 10.1016/j.neuroscience.2009.02.056

[236] Li Y, Zhou GM. Interneuron regeneration after ouabain treatment in the adult mammalian retina. Neuroreport. 2015;26:712–7 26164459 10.1097/WNR.0000000000000414

[237] Iseli HP, Wenzel A, Hafezi F et al. Light damage susceptibility and RPE65 in rats. Exp Eye Res. 2002;75:407–13 12387788

[238] Keller C, Grimm C, Wenzel A et al. Protective effect of halothane anesthesia on retinal light damage: inhibition of metabolic rhodopsin regeneration. Invest Ophthalmol Vis Sci. 2001;42:476–80 11157886

[239] Mishra A, Das B, Nath M et al. A novel immunodeficient NOD.SCID-*rd1* mouse model of retinitis pigmentosa to investigate potential therapeutics and pathogenesis of retinal degeneration. Biol Open. 2017;6:449–62 28258056 10.1242/bio.021618 PMC5399550

[240] Falasconi A, Biagioni M, Novelli E et al. Retinal phenotype in the rd9 mutant mouse, a model of X-linked RP. Front Neurosci. 2019;13:991 31607844 10.3389/fnins.2019.00991 PMC6761883

[241] Ghosh S, Liu H, Yazdankhah M et al. βA1-crystallin regulates glucose metabolism and mitochondrial function in mouse retinal astrocytes by modulating PTP1B activity. Commun Biol. 2021;4:248 33627831 10.1038/s42003-021-01763-5 PMC7904954

[242] Chen N, Li Y, Huang N et al. The Nrf2 activator MIND4-17 protects retinal ganglion cells from high glucose-induced oxidative injury. J Cell Physiol. 2020;235:7204–13 32020639 10.1002/jcp.29619

[243] Koo T, Park SW, Jo DH et al. CRISPR-LbCpf1 prevents choroidal neovascularization in a mouse model of age-related macular degeneration. Nat Commun. 2018;9:1855 29748595 10.1038/s41467-018-04175-y PMC5945874

[244] Holmgaard A, Alsing S, Askou AL et al. CRISPR gene therapy of the eye: targeted knockout of Vegfa in mouse retina by lentiviral delivery. Methods Mol Biol. 2019;1961:307–28 30912054 10.1007/978-1-4939-9170-9_19

[245] Fujikawa K, Iwata T, Inoue K et al. VAV2 and VAV3 as candidate disease genes for spontaneous glaucoma in mice and humans. PLoS One. 2010;5:e9050 20140222 10.1371/journal.pone.0009050 PMC2816215

[246] Shinozaki Y, Kashiwagi K, Namekata K et al. Purinergic dysregulation causes hypertensive glaucoma-like optic neuropathy. JCI Insight. 2017;2:e93456 28978804 10.1172/jci.insight.93456 PMC5841869

[247] Williams PA, Harder JM, John SWM. Glaucoma as a metabolic optic neuropathy: making the case for nicotinamide treatment in glaucoma. J Glaucoma. 2017;26:1161–8 28858158 10.1097/IJG.0000000000000767 PMC5854489

[248] Williams PA, Harder JM, Foxworth NE et al. Vitamin B3 modulates mitochondrial vulnerability and prevents glaucoma in aged mice. Science. 2017;355:756–60 28209901 10.1126/science.aal0092 PMC5408298

[249] Kha CX, Son PH, Lauper J et al. A model for investigating developmental eye repair in Xenopus laevis. Exp Eye Res. 2018;169:38–47 29357285 10.1016/j.exer.2018.01.007

[250] Kha CX, Tseng KA. Developmental dependence for functional eye regrowth in *Xenopus laevis*. Neural Regen Res. 2018;13:1735–7 30136686 10.4103/1673-5374.238611 PMC6128050

[251] Langhe R, Chesneau A, Colozza G et al. Müller glial cell reactivation in *Xenopus* models of retinal degeneration. Glia. 2017;65:1333–49 28548249 10.1002/glia.23165

[252] Fischer AJ, Reh TA. Identification of a proliferating marginal zone of retinal progenitors in postnatal chickens. Dev Biol. 2000;220:197–210 10753510 10.1006/dbio.2000.9640

[253] Fischer AJ, Dierks BD, Reh TA. Exogenous growth factors induce the production of ganglion cells at the retinal margin. Development. 2002;129:2283–91 11959835 10.1242/dev.129.9.2283

