# Supplementary material for: Retina regeneration: lessons from vertebrates
Source: Oxf Open Neurosci. 2022 Aug 2;1:kvac012. doi: 10.1093/oons/kvac012 (PMC10913848; doi:10.1093/oons/kvac012)
Supplement: suppl_data_kvac012 [file suppl_data_kvac012.zip › REVIEWSS FOR OXFNSC-2022-006.docx]

REVIEWSS FOR OXFNSC-2022-006.R1 Retina Regeneration: lessons from vertebrates

Original submission:

Reviewer 1

Review Article: Retina Regeneration: lessons from primitive vertebrates 

The review is informative and detailed encompassing many aspects of retina regeneration, an extremely interesting and relevant topic, in different species and classes of vertebrate animals. 
I particularly found that immune response regulation of retina regeneration is very interesting and conveys new insights in adult retina regeneration. 
However, the review is too extensive and a considerable part of the information can be found in different reviews that have been published before. Maybe a shorter and more accurate description of the most recent findings could be more informative without the need of unnecessarily repeating common knowledge. In this regard, the introductory part is too long. 
Orthography and grammar should be carefully corrected throughout the manuscript (there repeated words, lacking verbs, bad choice of words, and sometimes metaphoric language like “RPE resurrection”). 
There is too much information and sometimes is not clearly communicated making difficult to understand its relevance for readers that have not read all the original studies.

The reviewer has many comments and concerns about interpretation issues of experimental evidences (too subjective sometimes): 

I believe that calling primitive vertebrates to fish or amphibians or any other vertebrate or invertebrate species which are not mammalian vertebrates is misleading because no present species is primitive. Extinct ancestors of present species are primitive. Modern species of fish and amphibians actually are as evolved as any other species living at present on earth.  

Abstract: 
The Abstract is not well written and is misleading about the different regenerative mechanisms in teleost fish and amphibians. It is also not so accurate in differentiating growth mechanisms from regenerative ones. 
This sentence should be rephrased: “Soon after injury the Müller glial cells of the retina reprogram to for a proliferating population of Müller glia derived progenitor cells (MGPCs) capable of differentiating into various retinal cell type and Müller glia. “ 

- “Various retinal cell type and Müller glia”. Indeed to all neural cell types and Müller glia, because Müller glia is a retinal cell type. Importantly, this is accurate for teleost fish. However, this is not true for amphibian retinas, where transdifferentiation occurs from RPE cells. “Anuran amphibians can regenerate the retina through differentiation of stem cells in the ciliary marginal zone (mechanisms that sustain growth of retinal tissue in physiological conditions or accelerated growth after injury that contribute to retina regeneration) and particularly, through transdifferentiation of the retinal pigmented epithelium (regeneration)”. So, this is not well explained and may be misleading to non-experts in retina regeneration.  

Introduction  
Page 1. Line 27. “Like the rest of the CNS, the retina also has limited regenerative potential. “
The retina in mammalian species has no regenerative potential as far as I know in physiological or pathological conditions except that someone artificially manipulates proneural (pluripotency) gene expression. Anyhow, it is not clear whose retina has limited regenerative potential and the meaning of this sentence is somehow confusing. 
Lines 39-41. “The regenerative capability of primitive vertebrates is significant and complete mainly because of their preference over the slow regeneration compared to the faster wound healing. The mammals prefer the opposite which restricts their regenerative capability.”

I do not understand the meaning of this paragraph. I do not think that talking about preference is a good choice of words to refer to an evolutionary and adaptive process. To heal vs. regenerate I am sure is not dependent on the animal´s will. Moreover, I do not think this might be true, even if metaphorically speaking, since the strategy of healing over regenerating does not seem very wise. Healing is closing a wound but implies a permanent visual impairment. So, authors also meant that fish and frog‘s retina does not heal? Indeed, they not only heal their retinas but they conserve tissue function and visual capabilities throughout regeneration, and they do so in a relatively short period of time. 
The Introduction describes under the subtitle “Retinal Architecture” (lines 46-84) very classical and known vertebrate retina structural features (morphology, cell types, synaptic connections, etc) that can be found in any book. I do not think this is necessary. 
Lines 86-87. Retinal injury can be thru mechanical, chemical or light-induced methods (Figure 2). 
Several orthographic errors need to be corrected. 
There are other several experimental ways such as genetic ablation of specific cell types for injuring the retinal tissue to induce regeneration.

**Line 88. Mechanical injury is one the oldest and most feasible methods to study whole retina regeneration as it ensures uniform damage to all retinal layers.**
Correction of some orthographic errors is needed. 
I do not think mechanical injury is the most feasible method or even the oldest one. Light injury (even laser) and chemical injury are also classical and old methods for retina injury. The second part is not right either because it depends on the mechanical injury which may or may not be designed to cause uniform damage. Also, injury may not result so uniform. Well regulated chemical damage can also be uniform. Many times is also important damaging only one layer or the outer vs. the inner retina, which has been very informative. 
Description of injury paradigms is excessively long and it does not convey very new information for the field. 
**Lines 158-163.** In these paragraphs interpretation of the regenerative process and the stem cell population involved is confusing. I think authors mixed retinal growth and regeneration by talking altogether about the different classes of vertebrate and particular mechanisms. 

**Lines 165-166. In frogs, most of the retina forms during the tadpole stage from CMZ, with only marginal growth had happened during embryonic development [59]. In zebrafish, stem cells of CMZ divide asymmetrically in the radial axis, adding concentric rings of new cells [60].**
- What happens in frogs and in fish gets mixed up. It is not at all clear. 

**170- 175. The inability of stem cells in chick CMZ to generate all types of neurons may not be intrinsic to progenitors. Still, it could be due to local factors, which could be overcome by exogenous regulatory molecules [62, 63].**

- This is again confusing. Authors are talking about physiological processes or the way we can manipulate animal´s retinas to explore stem cell potential to give rise to all retinal types? Post hatch addition of bipolar and amacrine cells from CMZ progenitors in chicks for a month…is this a physiological process or is a result of artificially inducing CMZ progenitors to differentiate? It seems weird that only two types of inner neurons are peripherally added to the already formed retinal tissue (pre hatch). How do these new cells get connected to photoreceptors and ganglion cells? 

**Lines 175-176.** Evidences in mice about cell differentiation from CMZ stem cells have been artificially induced or naturally occurring? Are authors talking of embryos or postnatal animals?
**Lines 177-179. However, study in Xenopus tropicalis have shown that CMZ participates in complete retina regeneration after total retina removal [66].**
- This is again confusing. CMZ is part of the retina that has been totally removed, so, CMZ must be formed (regenerated) through RPE transdifferentiation. So, CMZ stem cells once formed may participate in retinal growth. I am not sure this is regeneration.

**Lines 186-187. Transdifferentiation requires interaction between the connective tissue and RPE which is choroid in newt while a fragment of the neural retina in embryonic chick [67].**

- The meaning of this sentence is completely unclear. It should be rephrased. 

**Line 190 - RPE has limited regenerative capability in adult fish and does not contribute to retina regeneration [71].**
However, RPE completely regenerates in adult fish. It is not directly involved in retina regeneration although evidences indicate that it releases important factors. 

**Lines 191-192. The avian embryos do not regenerate the retina spontaneously, but growth factor treatments (i.e. fibroblast growth factor, FGF) [72] or lin28 [73] are known to induce RPE transdifferentiation in them.**
So, the first part of the paragraph is misleading, because it states that RPE transdifferentiation “occurs” in avian embryos to regenerate the whole retina. Indeed, it “does not occurs” unless RPE cells are artificially induced and, as a result of the manipulation, retinas do not regenerate accurately or functionally. In my opinion the text is contradictory and confusing. 
**Lines 209-213. In response to retinal damage, Chick Müller glia enter the proliferative state and
express proneural genes [81].** 
**The reprogrammed Müller glia were sustained for a long time, expressing undifferentiated cell markers [79, 87]. The chick Müller glia, even after attaining
progenitor-like characteristics, majority of them do not produce neurons probably due to non-attainment of mature retinal progenitor cell identity [61].**

This paragraph is cryptic. Reprogrammed Muller glia state was sustained for a long time? What does it mean? Might it be part of a healing process? It does not seem to be part of a regenerative process (naturally occurring after injury). “Probably due to non-attainment of mature retinal progenitor cell identity” I cannot understand what the biological meaning of this last sentence is. 

**Lines 215-217. Without these manipulations, mouse Müller glia do respond to retinal damage, by migrating and expressing progenitor and cell cycle-specific markers but do not enter the cell cycle [90].**

So, from this paragraph and the description above, it is not at all clear which is the difference between mouse and chicken retinas regarding Müller glia progenitor gene expression and cell-cycle markers (Müller glia activate as a multipotent progenitor? ) and the lack of regeneration in both species of vertebrate. Do Müller glia in chicken retina proliferate (mitotically divide) without manipulations in response to in vivo injury? But they do not differentiate in neurons? Descriptions should state these differences more clearly. 

**Line 231 - rod and cone cells regenerate from slowly dividing neurod expressing progenitors with their origin in ONL [97].**
- NeuroD or neurod (in italics) expressing progenitors.

**Lines 248-270. These progenitors resemble those present during embryonic development stages; hence, responding cells seem to follow "winding the clock back" during retina regeneration.**
It should be clarified that this is not a demonstrated fact. This is a hypothetical point of view (interpretation) and other researchers in the field believe regeneration does not recapitulate embryonic development. For instance, tissue and cellular environments (and signalling) around progenitor Müller glia and multipotent proliferating progenitors as well as the epigenetic conditions are very different from embryonic tissue environments. In the regenerating tissue there are surviving and dying mature cells of different types whereas in the embryo environment all cells are undifferentiated progenitors or, later on, immature recently differentiated neurons (GC) but not yet Müller cells, and neurons are not still forming part of mature networks or interacting with glial and endothelial cells.  
If “chicks Müller glia proliferate after a retinal injury, but only a few differentiate into neuronal cell types consisting mainly of interneurons” this process does not seem to recapitulate embryonic development at all. So, is this because adult chick retina does not naturally regenerate or because regeneration and development are indeed different processes? 

**Lines 272-288.  Evidences regarding cancer development and regeneration relationship are not very convincing and the explanation given is confusing. It might occur some coincident signaling or transcription factor expression but it does not mean they are similar or mechanistically related processes. Moreover, after injury in zebrafish, TGβ signalling is first positively and later on negatively related to progenitor cell activation during retina regeneration. It is not clear from this paragraph if EMT (lines 285-288) or the opposite mechanism MET is related to progenitor Müller glia activation during regeneration (lines 276-278).**

Lines 309-313. The mice Müller glia express these transcription factors at the resting stage, which are downregulated soon after retinal damage but get upregulated to match the resting Müller glia at later stages [81]. These NFI factors maintain mice Müller glia quiescence, preventing transition to progenitor-like state and neurogenesis during development and as well post retinal damage [81, 120].

Is this a corroborated fact? Should not it be written as a hypothetical interpretation of partial evidences? I think this is described like if the whole history or the whole mechanism has been revealed. NFI factors are maybe involved in mammalian Müller glia quiescence but many other signalling pathways could be involved as well as other mechanisms. 

**Lines 313-314. Injury induced inflammation also plays a vital role in postnatal chick retina regeneration, where it activates reprogramming of Müller glia.**

I really do not understand and cannot tell if postnatal chick retina can or cannot regenerate by reading this review. Sometimes the authors state chick retina regenerates from progenitor Müller glia and sometimes they show evidences they cannot naturally regenerate. Evidences showed indicate that retinas regenerate only partially by generating interneurons, if they are experimentally induced (treated or manipulated) to do so. This is also confusing. 


**Line 341. Molecular basis of retina regeneration:**

This whole section is very informative but it needs to be carefully revised: 

For instance, but there are other sentences as well that needs correction:  

Lines 351- 353. “In zebrafish, the photoreceptor damage upregulates mmp9 in dividing Müller glia and photoreceptor progenitors, which TNF-α also induces even in intact retinae.” 

Lines 369-371. “ It is interesting to note that the anti- proliferative TGF-β signalling in mammals often turns pro-proliferative during the cancerous condition and is also during pro-proliferative pro-proliferative zebrafish retina regeneration.”

- Both sentences should be rephrased for a better understanding of their meaning. 

Moreover, in this section that deals with specific signalling pathway role in regulating progenitor Müller glia activation after injury for retinal repair several evidences are repeated from the text described above in different sections. For the sake of clarity, I think repeated information should be first consistently described throughout the manuscript and summarized and described in only one section to avoid unnecessary repetition, which could help reducing manuscript extension. 

Lines 396-398. Stab wound injury in zebrafish induces mTOR signalling and is essential for
dedifferentiation and proliferation of Müller glia and MGPCs by activating regeneration-
associated lin28a, ascl1a, cytokines and cell-cycle regulators [149].

I do not quite understand the difference between dedifferentiation and proliferation of Müller glia and MGPCs... Are the authors implying that these are two different populations of Müller glia? If so, it should be explained if not, the sentence should be corrected. 

Lines 399-400. “Also, the microglia/macrophage-mediated inflammation is a key regulator of mTOR in the Müller glia enabling mTOR-mediated retina regeneration retina regeneration [149].”

There is another section describing inflammation effects. The information and evidences should be reordered and consistently placed together throughout the manuscript under the corresponding subtitles or subsections. 

Lines 402-403.  
RPE resurrection? 
mTOR pathway probably belongs to immune and inflammatory regulation of retina regeneration section. 

Lines 448-450. “In the injured zebrafish retina, the Wnt signalling is triggered via an early
pan-retinal Ascl1a-Insm1a-Dkk axis which restricts back to the site of injury until the regeneration is completed [167]”.

I do not understand the meaning of this sentence. What does it mean “which restricts back to the site of the injury”. Please, could the authors rephrase it.  

Lines 512-513. The inability of mice Müller glia neural regeneration is due to STAT directed binding of Ascl1 to inappropriate targets [182].

This sentence is also pretty difficult to understand. There are many other signals throughout the review that explain “by themselves” the inability of mice retina to regenerate or Müller glia to proliferate and give multipotent progenitors to repair the retina. These should be considered all together otherwise it seems that each factor is described as the most relevant one in each section instead of a combination of factors and tissue conditions. 

Lines 522-524. In the chick retina, tet methylcytosine dioxygenase (TET3) facilitates DNA demethylation and RPE reprogramming, even in the absence of external growth factors [184].

Many times throughout the manuscript is not easy to understand if authors are talking about adult or embryonic retinas, particularly in chicks. The same consideration should be taken into account when authors are describing induced (manipulated) retinal proliferation and neurogenesis or the natural regenerative events occurring in amphibian and fish species.  

Line 539 . But the regeneration in mice retina seems to be more complex.
What do the authors mean? There is not natural occurring regeneration in mice. Something that does not occur cannot be more complex… So, hypomethylated states of regeneration-related gene promoters (such as oct4) in quiescent Müller glia do not seem a suited mechanism to explain the lack of retina regeneration in mice. Is this what the authors mean? Again, the whole paragraph is not clear. 

Lines 576-577. “The levels of some miRNAs get downregulated post retinal injury, suggesting
their involvement in maintaining a quiescent state or inhibiting Müller glia reprogramming.”

I do not agree with the meaning of this sentence. miRNAs downregulation could not be causally related in maintaining the quiescent state of Müller glia. Injured cells might stop other physiological functions (not related to survival, regeneration or reprogramming) so, these miRNAs could be related to functions of Müller glia or neural cells which are not fundamental or necessary any longer in the injured environment. 

Lines 596-598. Further, the overexpression of Ascl1 along with repression of histone deacetylase
[89] or inhibitor of STAT-signalling [182], made the mammalian retina more congenial for
regeneration.

What does it mean more congenial for regeneration? Rodent retina can be induced via genetic and epigenetic manipulations to proliferate and differentiate certain types of neural cells, up to what degree? Do proliferative cells stop growing tissue? Is there excessive growth that forms tumors? Do retinal cells assembly in neural networks that are functional? Is this related to regeneration or some forms of cancer induction?

Lines 602-603. “In land animals, the faster wound healing-preference over the slow regeneration could probably to avoid the risk of infection …”

I do not agree with this hypothesis. Aquatic animals do heal fast enough and also regenerate their retinas (avoiding losing function) and they have also to dodge infections if they want to survive (Why do the authors think a watery environment would be less risky for infections?).  

“…or due to the ability to lead a normal life despite having a compromised organ structure…”
What exactly do the authors mean by this part of the sentence? 

Lines 606-607. This ability to regenerate early during development could also attribute to the less complex epigenetic landscape facilitating gene expression.

….. could also be attributed to the less repressive epigenetic environment…

Reviewer 2

The review is very interesting as it describes recent advances in retina regeneration research using different models of primitive vertebrates. These studies may be useful to develop strategies for promoting regeneration in mammals. 
The review is organized into different sections that include information on the (i) architecture of the retina, (ii) the paradigms of injury, the cellular mechanisms involved in the regeneration processes, (iii) the homology of these processes with cancer, (iv) the immune response in the regeneration of the retina, (v) the molecular basis of retinal regeneration and a last paragraph in which the authors discuss about the mammalian regeneration. 

I have some observation about the text that could improve the manuscript: 

-I suggest including a conclusion at the end of each section. In some cases, it seems to be a list of findings, but the relevance of the review is to summarize and discuss the current information and try to answer, what is all that information telling us?

-In the section “Injury Paradigms” I suggest clarifying what types of paradigms are used in the different animal models.
At the end of this section there should be a final conclusion from the author about the 
relevance of the different paradigms used.

-Under the title “Regeneration cascades”, the authors describe the cellular mechanisms involved in the regeneration process (transdifferentiation, dedifferentiation, reprograming) . I consider it should be more appropriate changing the title of this section since the current title refers to cascades, a word that is usually used to describe molecular signaling mechanisms rather than cellular processes.

-In the section Molecular basis of retina regeneration, I consider that it is important to better organize the information by animal species. I suggest including a conclusion at the end of the section relating the pathways.

-I suggest carefully revising the text as there are some typos and some sentences that are not so clear.
i.e. Line 159, Fogs should be frogs

-I consider important to define abbreviations the first time they appear in the text.
i.e. RPE appears on line 140, but is defined on line 182.
Line 161, CMZ should be defined correctly the first time it appears

-Some sentences should be re-written because are confuse or repetitive
i.e. Line 180 "After removing the whole retina, retina regenerations....." (Avoid repetition)
i.e. Line 186 "Transdifferentiation requires interaction between the connective tissue and RPE which is choroid in newt while a fragment of the neural retina in embryonic chick". This sentence is confused.
-There are some redundant phrases :
i.e.:Line 189: "RPE has limited regenerative capability in adult fish and does not contribute to retina regeneration"
i.e.: Line 211 “The chick Muller glia, even after attaining progenitor-like characteristics, majority of them do not produce neurons probably due to non-attainment of mature retinal progenitor cell identity”

-Some molecules or transgenic animals cited in the text must be introduced briefly so the review can be accessible to readers who are not in the field. 
i.e.: line 191 “ The avian embryos do not regenerate the retina spontaneously, but growth factor treatments (i.e. fibroblast growth factor, FGF) [72] or lin28 [73] are known to induce RPE transdifferentiation in them” I suggest to introduce which type of molecule Lin28 is.
Line 205 “ In 2006, the use 101a1T:GFP transgenic fish ……” I suggest to give a brief description of this animal.
Line 230: “ In the diabetic model of zebrafish with pdx1 homozygous mutations” I suggest to give a description of this animal.
Line 268: “…. Differentiating into different neuronal cell types.” Repetition
Line 285: “Two factors ZEB1 and ZEB2….” Which type of factors are, give some relevant information about them, which is their role in cancer development.
-I also suggest using homogeneous nomenclature for genes and proteins mentioned throughout the manuscript
Line 338: Mmp9, line 349: mmp9
Line: 186 Sox2, line 374: sox2
Line 309-311: define NFI (Nuclear Factor I)
Line 327: define il34: Interleukin 34 (Il34)

REVISION:

Reviewer 1

The majority of the reviewer´s concerns have been met by authors´ corrections. Many grammatical and orthographic errors have been detected and corrected across the text. I believe the majority of the reviewer´s concerns have been satisfactorily explained and several aspects have been clarified throughout the text improving the understandability and informative value of the article. I think the manuscript has been greatly improved in terms of description clarity and topic organization. The addition of Tables and Figures has greatly improved the understanding of the particularities of all the species described in the different vertebrate classes. 

There are just a few issues that still need to be clarified or better expressed. Please, see the following paragraphs:
1) When authors describe developmental signaling pathways and transcription factors -particularly from line 594 and the following paragraphs- including developmental signaling pathways such as Hipo, mTor, Shh, Wnt-beta catenin-, MAP-ERK; Jak/Stat, etc, they described their importance for retinal regeneration in the adult retina of zebrafish. However, when they are comparing with chick, mouse or rat retinas it becomes still confusing whether they are talking about injury-induced regeneration process in embryonic retinas, or they are talking about the developmental process in itself or even postnatal stages (growth? Injury and/or gene /factor induced regeneration or neurogenesis?). 
Authors should include: “in the embryonic chick retina during development” or “in the embryonic chick retina during regeneration after injury”, “regeneration in the chick embryo retina after induction with FGF2”. Likewise for mammalian species. It is otherwise difficult to understand the information given without the need of looking for the original studies referenced in the manuscript. 

2) Page 3, line 77: primitive vertebrates should be replaced by cold blooded vertebrates or better yet: ectothermic vertebrates. I really believe we should not use “lower or primitive” (as I have mentioned before). 
3) LINES 76-80 and LINES 829-834. I still have serious doubts about the explanation/hypothesis given by authors of the aquatic vs. terrestrial environments and healing with loss of function vs. regeneration (which is also healing with tissue morphology and functional conservation). Furthermore, as far as I know not all aquatic fish can regenerate their retinas or brain structures (at least partially). Some teleost fish have been studied and/or described to do so as well as some amphibians. So, I do not think all aquatic fish (as a Class with a lot of modern species) follow the strategy of slow regeneration against quick healing as terrestrial animals do (according to the authors). So, it is still not very convincing. 
Anyway, if authors consider it as a plausible hypothesis it should be clearly stated as a highly hypothetical cause of the reduced regenerative capability (particularly of the CNS) in terrestrial vertebrates and hence consider what happens with aquatic animals that heal quickly instead of regenerating.
4) Answer: We have modified the above sentences to: ‘In frogs, most of the retina forms during the tadpole stage from CMZ, with only marginal growth had happened during embryonic development [59]. The stem cells residingin CMZ also contribute? to zebrafish retinal growth by dividing asymmetrically in the radial axis and adding concentric rings of new cells [60].’

Concern. …”‘In frogs, most of the retina forms during the tadpole stage from CMZ, with only marginal growth occurring or happening during embryonic development”… 

It is still unclear what do the authors mean. How can the retina “grow” in the embryonic development (early stage) and “form” during the tadpole stage (later stage)? What do authors mean by also contribute in the zebrafish sentence? 
In zebrafish, retina partially forms during embryonic development and continuously and primarily grows from CMZ during larval, juvenile and adult stages through the animal´s life. As in frogs the majority of the mature retinal tissue forms and grows from the CMZ. Anyway, I cannot understand whether authors are describing a difference between frogs and zebrafish or a similarity? In both vertebrate classes adult retina forms almost completely from CMZ stem cells. In zebrafish CMZ stem cells are not just “also contributing” to retina growth… 

5) We have elaborated these sentences for clarity, like this: ‘Chicks' eyes continue to grow for about a month after hatching with retinal development by cells within the CMZ. However, unlike amphibians and fish, only amacrine and bipolar cells are added to the chick retina under normal physiological conditions (without external factors). 

-I suggest that authors may perhaps add a sentence regarding the absence of evidences (knowledge) regarding new generated interneuron cell integration to the mature retinal tissue in post hatch chicks. 
6) Lines 280-284. We have reframed this sentence as: ‘Transdifferentiation requires interaction between the connective tissue and RPE. In newt choroid acts as a connective tissue, while in embryonic chick it is a fragment of neural retina [67]. 

Transdifferentiation requires interaction between the connective tissue and RPE. In the newt, the choroid acts as a connective tissue, while in the embryonic chick it is a fragment of the neural retina [52]. 

It is not clear in which species transdifferentiation from RPE requires interaction with connective tissue itself not being the choroid (after reading table 2 it is still more confusing because according to the text I thought it was in frogs or other amphibians except Newt). Transdifferentiation requires interaction of the RPE with connective tissue … (Which connective tissues?). The authors described the choroid in Newt which “acts as a connective tissue” or a fragment of neural retina in chicks”. Indeed, the choroid is a highly vascular connective tissue or vascular tissue. This should be corrected either in the text or in table 2 line 26, because in this table authors have stated that transdifferentiation occurs via interaction with the choroid layer in Amphibians (which includes Newt, frogs, Xenopus, etc). Moreover, the neural retina is not a connective tissue… So, please clarify.
In text citation (52) is different to the one informed in the answer to the reviewers (67). Please revise it.
7) Line 271 ... “The embryonic stages of anuran amphibians and avian embryos can regenerate their retina”
I think it may be better this way: “Anuran and avian embryos can regenerate their retina…”.

8) Lines 287-288 Please, correct this sentence. 
“…an RNA binding protein which is important pluripotency inducing factor involved in reprogramming,) [59].” Change to … “an RNA binding protein which is an important pluripotency factor involved in reprogramming.”

9) Lines 327-328… Müller glia-derived progenitors, which migrate to ONL express Crx (cone -rod homeobox), are the retinal progenitors generating rod photoreceptor lineage [63].
Change to: Müller glia-derived progenitors, which migrate to ONL and express Crx (cone -rod homeobox), are the retinal progenitors generating rod photoreceptor lineage [63].

10) Lines 421-422. Please, revise the grammar in this sentence. 
A recent study with transcriptomic profiling of chick, zebrafish, and mice retina suggests mice Müller glia quiescence due to nuclear factor I (Nfia/b/x) and Sox5.

11) Lines 472-473 Upon retina damage, dyeing retinal neurons…
Should be corrected to “dying retinal neurons”. 

12) line 520 “The Sox2 is a crucial player in zebrafish Müller glia reprogramming”… Please, change to: “Sox2…” or “the Sox2 transcription factor…”

13) Lines 523-524. “…alongside this post-retinal damage, sustained Sox2 expression allows a regenerative response similar to zebrafish…”. Please rephrase. 

14) Lines 558-560. Retinal damage triggers mTOR signaling in activated Müller glia and its inhibition impairs MGPCs proliferation even in EGF2 treated retinae. Inhibition of the mTOR pathway also surpasses the MGPC-promoting effects of glucocorticoid, sonic hedgehog, and wnt signaling in the embryonic (?) chick retina [137].

15) Please, correct Table 2 because some lines are displaced (For instance see lines 34-36). 

Reviewer 2

The review contains very complete and detailed information on the retinal regeneration process in different species of vertebrate animals.
I consider that the new version of the review has substantially improved both the content as well as the spelling and grammar that makes it much easier to read.
In the new version of the work, the information is better organized, which allows a better understanding of its content. The authors have included integrative conclusions in each section that highlights the most important events on the regeneration process in the different species analyzed.
In the same way, the inclusion of the 2 tables comparing the paradigms of retinal injury and the mechanisms involved in retina regeneration facilitates the comparison of the different processes in the analyzed species.
